# A novel, non-neuronal acetylcholinesterase of schistosome parasites is essential for definitive host infection

**DOI:** 10.3389/fimmu.2023.1056469

**Published:** 2023-01-31

**Authors:** Patrick J. Skelly, Akram A. Da’dara

**Affiliations:** Molecular Helminthology Laboratory, Department of Infectious Disease and Global Health, Cummings School of Veterinary Medicine, Tufts University, North Grafton, MA, United States

**Keywords:** acetylcholinesterase, host-parasite interaction, non-neuronal, tegument, schistosoma

## Abstract

Schistosomes are long-lived parasitic worms that infect >200 million people globally. The intravascular life stages are known to display acetylcholinesterase (AChE) activity internally as well as, somewhat surprisingly, on external tegumental membranes. Originally it was hypothesized that a single gene (SmAChE1 in *Schistosoma mansoni*) encoded both forms of the enzyme. Here, we demonstrate that a second gene, designated “*S. mansoni* tegumental acetylcholinesterase, SmTAChE”, is responsible for surface, non-neuronal AChE activity. The SmTAChE protein is GPI-anchored and contains all essential amino acids necessary for function. AChE surface activity is significantly diminished following SmTAChE gene suppression using RNAi, but not following SmAChE1 gene suppression. Suppressing SmTAChE significantly impairs the ability of parasites to establish infection in mice, showing that SmTAChE performs an essential function for the worms *in vivo*. Living *S. haematobium* and *S. japonicum* parasites also display strong surface AChE activity, and we have cloned SmTAChE homologs from these two species. This work helps to clarify longstanding confusion regarding schistosome AChEs and paves the way for novel therapeutics for schistosomiasis.

## Introduction

Schistosomiasis is a parasitic disease caused by helminth parasites of the genus *Schistosoma* (1, 2). Schistosomiasis ranks among the most important infectious diseases globally, affecting more than 200 million people world-wide, and over 700 million people live at risk of infection (2–6). In sub-Saharan Africa alone, mortality is put at ~280,000 deaths per year, with tens of millions having chronic morbidity (4, 7, 8). Furthermore, extensive pathological changes associated with schistosome infection can affect multiple organ systems, including the liver, spleen and the gastro-intestinal tract, especially in individuals with longstanding or heavy infections (9). Three major species cause schistosomiasis in humans: *S. mansoni*, *S. haematobium* and *S. japonicum*. Schistosomes have complex life cycles. Larvae (cercariae) are released from infected intermediate, freshwater snail hosts. Upon encountering a human (the definitive host), cercariae penetrate the skin, transform into juvenile forms called schistosomula that migrate through the bloodstream to the liver where they mature into adult male or female parasites. The adults mate and females produce hundreds of eggs each day, some of which are released into the environment. In fresh water, the eggs hatch and give rise to free swimming larvae (miracidia) that seek and enter the snails to continue the life cycle.

In our laboratory, we study the molecular and cell biology of the schistosome tegument (skin) (10–16). The tegument of the intravascular life stages is a major site for host-parasite interaction, and proteins that make up the tegument represent potential therapeutic targets and vaccine candidates. Here we focus on schistosome tegumental acetylcholinesterase (AChE; EC. 3.1.1.7).

AChE, a widely distributed enzyme in animals, is best known for its ability to catalyze the breakdown of the neurotransmitter acetylcholine. This limits the interaction of acetylcholine with its receptors at cholinergic synapses. The immunolocalization of AChE in the neuromusculature of schistosomes, and the ability of anticholinesterase compounds to paralyze adult worms, are both consistent with a role for this enzyme in controlling neuromuscular activity in these parasites, as in other animals (17–20). Somewhat surprisingly, AChE has additionally been localized in the tegument of intravascular-stage schistosomes, and AChE activity has been associated with isolated tegumental membranes (17, 20–22). Indeed, more than 50% of total *S. mansoni* AChE activity can be detected at the surface of 24-h cultured schistosomula (23). The precise relationship between the tegumental and non-tegumental AChE in schistosomes has been unclear for many years. Earlier biochemical characterization suggested the existence of two different AChE enzymes differing in their solubility characteristics and quaternary structures (24, 25). One form, with a sedimentation coefficient of 6.5S, did not bind to heparin (26). Since this AChE is released from intact parasites by treating them with phosphatidylinositol-phospholipase C (Pi-PLC), it is considered to be the tegumental, host-interactive AChE (26, 27). A second AChE form had a sedimentation coefficient of 8S and bound heparin (26). In addition, in experiments with live parasites, the non-membrane permeable AChE inhibitor echothiophate (phospholine) selectively blocked the 6.5S enzyme but not the 8S form, suggesting that the 8S form is located internally (26). However, heterologous anti-AChE antibodies could not distinguish between two distinct schistosome AChE forms and a single identified AChE gene was proposed to encode both the internal (neuromuscular) and external (tegumental) enzymes (17). A gene encoding AChE was identified not just in *S. mansoni* (now designated SmAChE1) (28) but also in *S. haematobium* (17), S*. bovis* (28) and *S. japonicum* (29). However, not one of the proteins encoded by these genes is predicted to contain a GPI anchoring sequence, suggesting that none encode the surface form of AChE. A second AChE gene has been identified in *S. mansoni* (SmAChE2) but its reported sequence also lacks both a predicted GPI anchoring motif and a leader sequence (30). However, purified recombinant SmAChE1 and SmAChE2 do both cleave acetylthiocholine, and suppression of either gene diminishes the cholinesterase activity measured in extracts of gene-suppressed schistosomula compared with controls (30). Antibodies raised against recombinant fragments of SmAChE1 and SmAChE2 bind widely throughout the adult worms’ internal structures as well as in the tegument. However, given that both AChEs share multiple conserved motifs, it is likely that antibodies against one will cross react with the other. Finally, while both SmAChE1 and SmAChE2 are reported to immunolocalize on the tegument “surface” (30) the resolution by immunofluorescence microscopy is not sufficient to support this claim.

Here, we set out to identify and characterize the gene encoding the surface AChE in the three major schistosome species that infect humans and to determine if the previously characterized AChE genes encode the enzymes responsible for surface activity.

## Materials and methods

### Mice and parasites

Female, 6–8 week-old Swiss-Webster CD1 mice were purchased from Charles River and maintained under specific pathogen-free conditions at the animal facility of Cummings School of Veterinary Medicine, Tufts University. All experimental procedures involving animals were carried out in accordance with approved guidelines of the Institutional Animal Care and Use Committee (IACUC) of Tufts University and all animal work was done in the vivarium at Cummings School of Veterinary Medicine, Tufts University.

*Biomphalaria glabrata* snails, infected with *Schistosoma mansoni* (Puerto Rican NMRI strain) were obtained from the NIAID Schistosomiasis Resource Center of the Biomedical Research Institute (BRI), Rockville, MD, USA, and maintained in our laboratory. Cercariae, infectious larvae, were prepared by exposing infected snails to light for 1-2 h to induce shedding. Cercarial numbers and viability were determined using a light microscope.

Schistosomula were prepared from cercariae by mechanical removal of the cercarial tails *via* vortexing and subsequent Percoll purification, as previously described (31). Schistosomula were cultured in complete DMEM/F12 medium (Invitrogen) supplemented with 10% heat-inactivated fetal bovine serum (FBS), 200 U/ml penicillin and 200 μg/ml streptomycin (Invitrogen), 1 μM serotonin (Sigma), 8 μg/ml human insulin (Sigma), 0.2 μM triiodothyronine at 37°C, in an atmosphere of 5% CO_2_. Schistosomula numbers and viability were determined using a light microscope and the trypan blue exclusion test.

Adult male and female parasites were recovered by perfusion from Swiss Webster mice that were infected with 120 cercariae (*S. mansoni*) or 25 cercariae (*S. japonicum*) 7 weeks previously (32–34). Adult *S. haematobium* were recovered by perfusion of Golden Syrian hamsters that had been infected with 350 cercariae 12 weeks previously. In all cases, the mesenteric veins and the liver vasculature were examined for the presence of any parasites that were not washed out by perfusion. Both *S. japonicum* infected mice and *S. haematobium* infected hamsters were provided by BRI. Adult parasites were maintained in complete DMEM/F12 medium supplemented as described above. Schistosome eggs were isolated from livers of mice infected with *S. mansoni*, as previously described (34, 35).

### Measuring acetylcholinesterase (AChE) activity

AChE activity was measured at room temperature by the modified Ellman method using acetylthiocholine iodide (ATCh) as substrate (36). The reaction mixture contained 1 mM acetylthiocholine and 1 mM 5,5’-dithiobis(2-nitrobenzoic acid) (DTNB) in 100 mM sodium phosphate, pH 7.2, in a total volume of 200 µl. DTNB is a membrane impermeant reagent (37, 38). Total parasite lysates were prepared by homogenizing a specific number of male or female parasites in ice-cold 100 mM phosphate buffer, Ph 7.2. Experiments were carried out on parasite lysates or on live, individual male or female parasites, or groups of cercariae, schistosomula or eggs, in PBS or in clear, serum-free DMEM/F12 medium containing reaction mixture. There was no significant difference in the activity between PBS and clear medium and in all subsequent experiments clear medium was used with living worms. Absorbance at 412 nm was monitored over time (mostly every 5 mins) using a Synergy HT spectrophotometer (Bio-Tek Instruments, Winooski, VT, USA). Note that parasites remained in the wells during this assay, and their presence did not impact readings. In some experiments the acetylcholinesterase inhibitor BW284c51 (1,5-bis(4-allyldimethylammoniumphenyl)pentan-3-onedibromide, Sigma-Aldrich) was added at a final concentration of 100 µM. Some experiments were conducted using butyrylthiocholine iodide (BuTCh, Sigma-Aldrich, 2.5 mM), and not acetylthiocholine, as substrate.

### Cloning and characterization of the tegumental AChE (SmTAChE) of *S. mansoni*

Since initial studies showed that the surface AChE was not encoded by the known AChE gene (now called AChE1), this led us to search for another gene which could potentially encode the surface AChE. *S. mansoni* data bases (http://schistodb.net/, https://parasite.wormbase.org/index.html and http://www.genedb.org/Homepage/Smansoni) were queried for the presence of potential AChE sequences. Based on signature elements found in other acetylcholinesterases, several genes were identified including a strong candidate (ID: Smp_136690). All other early database hits displayed only modest sequence similarity over short distances and were filtered out on this basis. The Smp_136690 coding sequence was amplified using the following primers: SmAC2-F: 5’-TGACTATTTGGATACACTTATG-3’, and SmAC2-R: 5’-TCTATGAAGTCATTTTACAAGG-3’, designed from database information, and using AccuPrime Taq DNA Polymerase high fidelity (following 40 cycles at 95°C for 30 sec, 50°C for 30 sec and 68°C for 3 min), as per the manufacturer’s recommendations (Thermo Fisher Scientific). cDNA synthesized from total RNA (obtained from a mixture of adult male and female worms) was used as a template for the PCR. The amplified product was purified and sequenced at Tufts University Core Facility and the final clone was designated SmTAChE. RNA isolation and cDNA synthesis is described below under “SmTAChE gene expression analysis”. Sequence analysis using multiple tools (described below) was used to characterize potential AChEs.

### Cloning of the tegumental AChEs of *S. haematobium* and *S. japonicum*

Analysis of the *S. haematobium* genome at schistoDB.net resulted in the identification of a gene (KL250835) encoding a protein with a high degree of sequence similarity to SmTAChE (now designated MS3_0012973 at *WormBase Parasite*, https://parasite.wormbase.org/index.html). The following oligonucleotides flanking the predicted open reading frame (ORF) were used to amplify the cDNA using RNA isolated from mixed adult stage parasites: ShAC2-F: 5’-AATATTCTTTCTTCCTATTGACAATG-3’, ShAC2-R: 5’-ACATTTTCATCAATATAAAAAC-3’. The amplified coding DNA was sequenced and designated ShTAChE.

Analysis of the *S. japonicum* genome did not immediately reveal a gene encoding a protein similar to SmTAChE. However, extensive analysis revealed the presence of sequences encoding potential fragments of a SmTAChE homolog on two different contigs: Sjp_0045440 (now known to code for the first exon), and Sjp_0070510 (now known to encode the remaining 3 exons) (*WormBase ParaSite:* https://parasite.wormbase.org/index.html). To isolate the full predicted coding sequence, a rapid amplification of cDNA ends (RACE) experiment was performed using the 3’, 5’ SMARTer RACE kit, with mixed adult stage RNA and the following gene-specific primers, (following the manufacturer’s instructions, Clontech): SjAC2GSP-S1: CAGGCAGTGCTAATCTACAAGTATACAATGGTGC-3’ and SjAC2GSP-S2: GCTGAACATGTTGCTAGATTACCAAATGC, (for the 3’end), and SjAC2GSP-AS1: GCACCATTGTATACTTGTAGATTAGCACTGCCTG and SjAC2GSP-AS2: GCATTTGGTAATCTAGCAACATGTTCAGC-3’ (for the 5’end). Several fragments with the predicted size were amplified and sequenced. In this manner the full ORF, encoding what we now call SjTAChE, was identified, and this confirmed that the two contigs abut.

### *In Silico* analysis of the predicted tegumental AChEs

Several online tools were used to analyze the deduced amino acid sequence of the tegumental AChEs. For the prediction of GPI anchors and omega residues, the GPI-SOM (http://gpi.unibe.ch/) and the NetGPI (https://services.healthtech.dtu.dk/service.php?NetGPI) tools were used. Analysis of the signal peptide was performed using SignalP prediction software (https://services.healthtech.dtu.dk/service.php?SignalP-5.0). Analysis of potential N-glycosylation sites was performed using the NetNGlyc 1.0 Server (https://services.healthtech.dtu.dk/service.php?NetNGlyc-1.0). Multiple alignment analysis was conducted using CLUSTAL O (1.2.4) at http://www.ebi.ac.uk/Tools/msa/clustalo/. Phylogenetic analysis was performed at phylogeny.fr (http://www.phylogeny.fr/) (39–41) using default settings (Alignment: Muscle; Maximum number of iterations: 16; Curation: Gblocks; Phylogeny: PhyML; Statistical test for branch support: approximate likelihood ratio test (SH-like); Number of substitution rate categories: 4).

### Anti-SmTAChE antibody production

An SmTAChE-specific peptide, comprising amino acid residues K^328^ – E^346^ (NH_2_-KHRYDAVRKYLLPRYHKQE-COOH), was synthesized by Genemed Synthesis, Inc. (San Antonio, TX). Note that this peptide represents a sequence unique to SmTAChE and is not found in SmAChE1 (accession no. AAQ14321.1). A cysteine residue was added at the peptide’s amino terminus, to facilitate conjugation to bovine serum albumin (BSA). Approximately 500 µg of peptide-BSA conjugate in Freund’s Complete Adjuvant was used to immunize two New Zealand White rabbits subcutaneously. The rabbits were boosted with 100 µg of peptide alone in Incomplete Freund’s Adjuvant 20, 40, and 60 days later. Ten days following the last immunization, serum was recovered from both rabbits, pooled and anti-SmTAChE antibodies were affinity-purified using the same peptide at Genemed Synthesis.

### RNA interference

Schistosome parasites (adults and schistosomula) were treated with a synthetic siRNA (from IDT DNA Technologies) targeting either SmAChE1 (AF279461), or SmTAChE (OP018961) or no sequence in the schistosome genome (Control). Sequences of all siRNAs are provided in **Table S1**. siRNAs were delivered to parasites by electroporation, as described previously (31). Gene suppression was assessed post-treatment by comparing mRNA levels using reverse transcription quantitative PCR (RT-qPCR) and by comparative enzyme activity measurements in target versus control groups.

### SmTAChE gene expression analysis

The level of expression of both the SmAChE1 and the SmTAChE gene in different life stages of the parasite, and in parasites treated with gene-specific siRNAs, was measured by RT-qPCR, using custom TaqMan gene expression systems (Applied Biosystems, CA). First, RNA from different life stages was isolated using TRIzol Reagent according to the manufacturer’s instructions (Invitrogen). RNA was then treated with DNaseI to remove any genomic DNA, using a Turbo-DNase I kit (Ambion). cDNA was synthesized using 0.5 µg RNA, an oligo (dT)_12-18_ primer and Superscript III RT (Invitrogen). For developmental expression, triose phosphate isomerase (TPI) was used as a reference gene, as previously (31). Primer sets and reporter probes labeled with 6-carboxyfluorescein (FAM) were obtained from Applied Biosystems, CA (**Table S2**). All samples were run in triplicate and underwent 40 amplification cycles on a StepOne Plus Real Time PCR System. For relative quantification, the ΔΔCt method was employed (31). For RNAi experiments, the schistosome alpha-tubulin gene was used as the “within-stage” endogenous control, as earlier (13, 31).

### Pi-PLC treatment and western blot analysis

To monitor the expression of the SmTAChE protein, parasite samples were first homogenized in ice-cold lysis buffer (20 mM Tris-HCl, pH 8.0 containing 2% SDS). Protein concentration was determined using the Pierce Micro BCA Protein Assay Kit (ThermoFisher Scientific) according to the manufacturer’s instructions. GPI-anchored proteins were recovered from live male worms by *in vitro* incubation with phosphatidylinositol-phospholipase C (Pi-PLC) (Sigma-Aldrich), as previously described (42). Briefly, 20 freshly perfused male parasites were washed three times in DMEM and were then incubated at 37°C for 1 h in the presence of Pi-PLC (from *Bacillus cereus*) at 1.25 Units/ml. The supernatant was removed and concentrated at 4°C using a 10-kDa cut-off Pierce Protein Concentrators (ThermoFisher Scientific). Proteins were resolved by SDS-PAGE under reducing conditions and blotted to PVDF membrane. Membranes were blocked using Tris-Buffered Saline (TBS) containing 0.05% Tween 20 (TBST) and 5% non-fat dry milk. The membrane was then probed overnight at 4°C with affinity purified rabbit anti-SmTAChE antibody at 1:1000 dilution. After three washes in TBST, bound primary antibody was detected using horseradish peroxidase-labeled anti-rabbit IgG (1:5000, GE Healthcare). Signals were detected using ECL Western Blotting Detection Reagents (GE Healthcare). Western blot images were captured using a ChemiDoc Touch Imaging System (Bio-Rad).

### Immunolocalization of SmTAChE

Freshly perfused parasites were embedded in OCT compound and flash frozen in liquid nitrogen. Adult worm frozen sections (7 μm thick) were obtained using a cryostat and fixed in ice-cold acetone for 30 min at -20°C. Cultured schistosomula (7 day) were fixed in 4% paraformaldehyde for 20 min at room temp. Parasites/parasite sections were washed three times in PBS before being incubated with 1% BSA in PBS (blocking buffer) for 1h. The samples were incubated with primary, purified anti-SmTAChE, antibody at 1:100 dilution for 1h. After washing with PBST (PBS containing 0.05% Tween-20), parasites were then incubated with Alexa Fluor-488-anti-rabbit IgG (H+L, Invitrogen) diluted 1:100 in blocking buffer for 1h, as described (43). Samples were washed in PBS, mounted in Fluoromount and viewed using an inverted fluorescent microscope (TH4–100; Olympus, Tokyo, Japan) equipped with a Retiga 1300 camera (Q Imaging, BC, Canada).

### Immunogold labeling and electron microscopy

Freshly perfused adult parasites were fixed overnight with 2% glutaraldehyde in 0.1M cacodylate buffer at 4°C. The samples were then dehydrated in a graded series of ethanol, then infiltrated and embedded in L.R. white acrylic resin. Ultramicrotomy was performed using a Leica Ultracut R ultramicrotome and the sections collected on gold grids. Grids were immunolabeled in a two-step method as follows: the grids were conditioned in PBS for 5 min x 3 at room temperature, followed by the blocking of non-specific labeling for 30 min at room temp using 5% non-fat dry milk in PBS. After rinsing, the grids were exposed to primary antibody diluted 1:30 for 1h at room temperature, followed by washing in PBS and then incubated with secondary antibody diluted 1:30 (10 nm gold-labeled goat anti-rabbit IgG (H&L, GE Healthcare)) for 1h at room temperature, and finally rinsed thoroughly in water. Control parasite preparations were treated with secondary antibody alone. Grids were exposed to osmium vapor and/or lightly stained with lead citrate to improve contrast and were examined and photographed using a Philips CM 10 electron microscope at 80KV.

### Infection of mice with SmTAChE gene suppressed schistosomula

One-day-old cultured schistosomula were electroporated with siRNA targeting SmTAChE, or with control siRNA, or with no siRNA (**Table S1**). Parasites were maintained in culture for 4 days, then counted and resuspended in phenol-red-free RPMI medium. Approximately 1000 schistosomula were used to infect each mouse by intramuscular injection (100 µl total volume) into the thigh muscles, using a 1 ml tuberculin syringe and a 25G-1 needle (12, 44). Mice were perfused 6 weeks later, and worm burdens were determined (32–34).

### Statistical analysis

Data were assessed for normality using Shapiro-Wilk tests with GraphPad Prism 9.4. Bartlett’s test for homogeneity of variances confirmed the assumption of equal variances. The student’s *t*-test, one-way analysis of variance (ANOVA) with Tukey *post hoc* analysis and two-way ANOVA were used to compare the means between a target group and a control group using GraphPad Prism 9.4. P values less than 0.05 were considered significant.

## Results

### Schistosome parasites exhibit surface AChE activity

**Figure 1:**
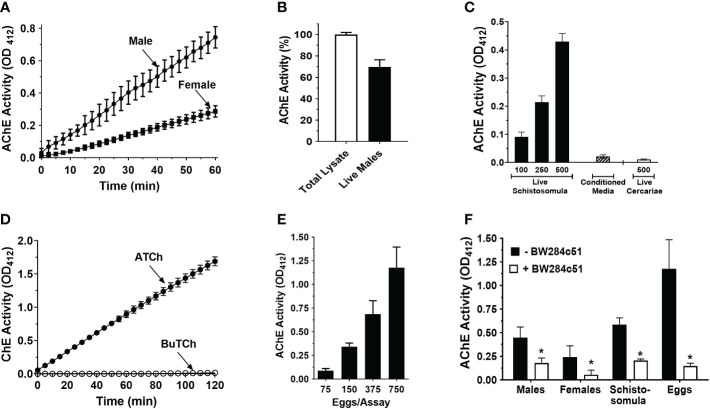
Acetylcholinesterase (AChE) activity (mean OD_412_ ± SD) in live *S. mansoni* parasites. **(A)** Acetylcholinesterase activity in individual live male (circles, n>10) or female parasites (squares, n>10). Male parasites display significantly higher surface AChE than females (two-way ANOVA, p<0.0001). **(B)** Surface AChE activity of individual live male parasites (at 60 min, black bar) compared to total AChE activity recorded in lysates of individual male parasites (white bar, set at 100%). **(C)** AChE activity in groups of live, 7-day-old cultured schistosomula (black bars) or in 24 h conditioned media (from 500 cultured schistosomula, hatched bar) or in groups of 500 cercariae (white bar), at 60 min (n≥3), **(D)** Cholinesterase (ChE) activity (mean OD_412_ ± SD) displayed by live schistosomula (groups of 1000, n=4) in the presence of acetylthiocholine (ATCh, 2.5 mM, closed circles) or butyrylthiocholine (BuTCh, 2.5 mM, open circles); no cholinesterase activity is detected when BuTCh is included as substrate. **(E)** AChE activity in groups of freshly recovered liver eggs at 60 min, (n>5). **(F)** Surface AChE activity of different schistosome life stages at 60 min, (1 male, 1 female, 500 schistosomula, or 750 eggs per assay, n≥4) in the presence (white bars) or absence (black bars) of the acetylcholinesterase inhibitor BW284c51 (100 µM final concentration; * t test, p<0.05).

As shown in **Figure 1A**, intact, individual live male or female adult *S. mansoni* cleave exogenous acetylthiocholine. The larger male worms exhibit significantly higher (>2X) surface AChE activity compared to females (p<0.001). When the AChE activity of total lysates of individual male worms is set at 100%, the surface AChE activity (i.e., that displayed by live individual worms) is approximately 70% of this value (**Figure 1B**). As shown in **Figure 1C**, live schistosomula also display measurable AChE activity, and the greater the number of schistosomula the greater the activity recorded. In addition, essentially no AChE activity is detected in clear, conditioned, serum-free culture medium where 500 schistosomula had been cultured for 24 hours (Conditioned Media, **Figure 1C**), However, no AChE activity is detected using freshly shed live cercariae (**Figure 1C**). Furthermore, no activity is observed when butyrylthiocholine is incubated instead of acetylthiocholine with live schistosomula, as indicated in **Figure 1D**. Similarly, no butyrylcholinesterase activity was detected when live adult parasites were used in similar experiments (data not shown). Schistosome eggs, freshly isolated from the liver tissue of infected mice, exhibit substantial AChE activity (**Figure 1E**). Once again, the greater the number of parasites in the assay, the greater the activity seen. As shown in **Figure 1F**, the surface AChE activity of all life-stages is significantly inhibited in the presence of the selective AChE inhibitor, BW284c51 (p<0.05 in all cases).

### Identifying the surface AChE enzyme

**Figure 2:**
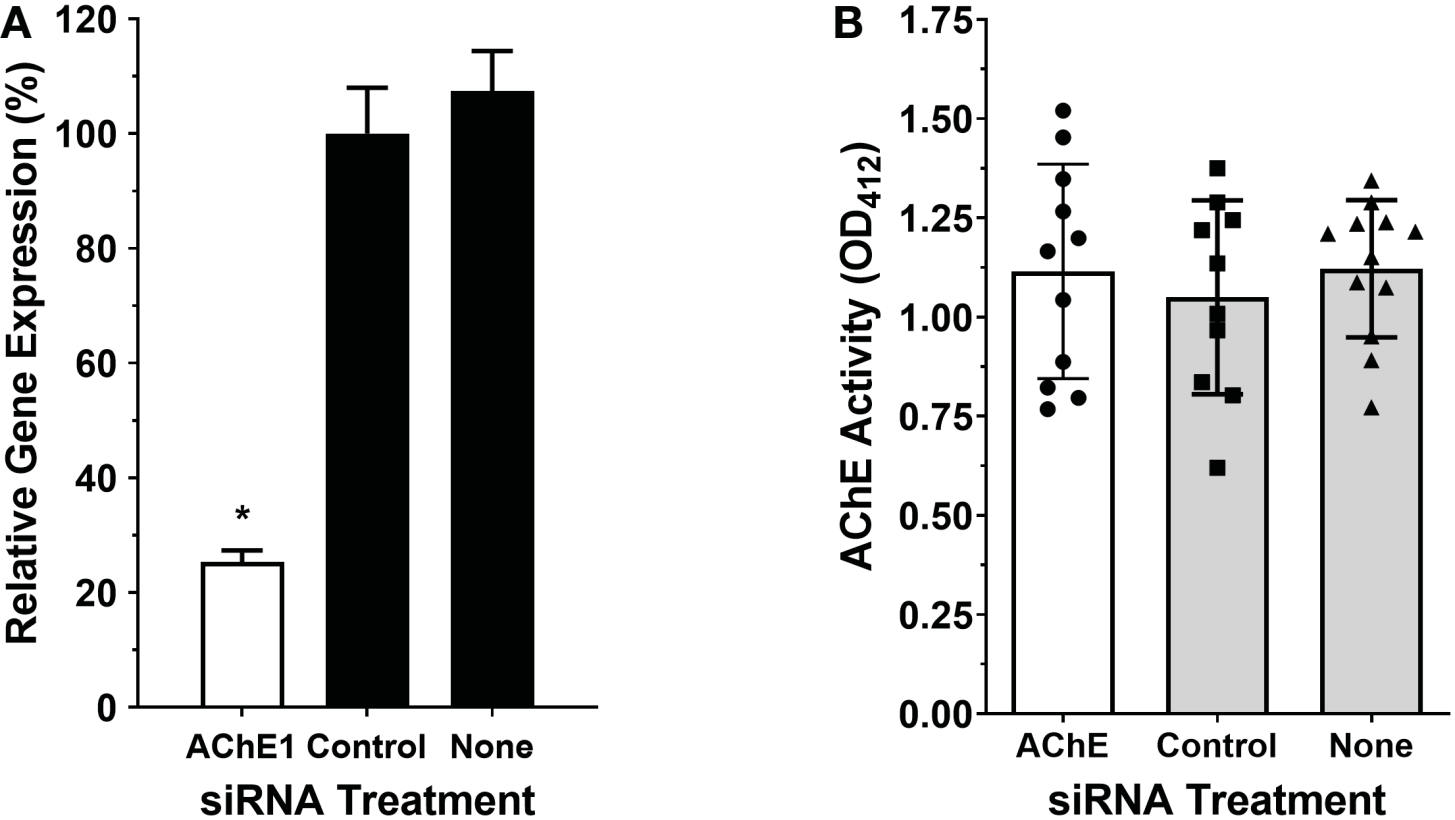
Suppression of SmAChE1 gene expression. Adult male parasites were treated either with an siRNA targeting SmAChE1, or with an irrelevant siRNA (Control) or with no siRNA (None), as indicated. **(A)** Relative expression of the SmAChE1 gene, measured by RT-qPCR seven days later. Treating parasites with siRNA targeting SmAChE1 significantly suppressed the expression of this gene (by ~ 70%; white bar, *p < 0.05 ANOVA) compared to controls. **(B)** AChE activity was measured in live individual male parasites treated with siRNA, as indicated. Mean AChE activity of the three groups of parasites did not differ significantly when tested 7 days post siRNA treatment. Each point represents data from an individual parasite, and the bars indicate the mean ( ± SD) for the group.

**Figure 3:**
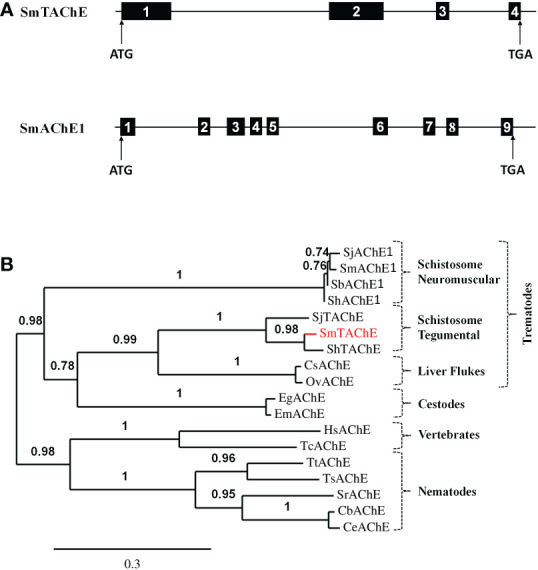
**(A)** Schematic representations of the SmTAChE (top) and SmAChE1 (bottom) genes. Black boxes represent the numbered exons and thin lines introns. The positions of the start (ATG) and stop (TGA) codons are noted. **(B)** Phylogenetic analysis of the following AChEs: *S. mansoni* SmTAChE, (Accession no: OP018961, red), *S. haematobium* ShTAChE, (OP018962), *S. japonicum* SjTAChE, (OP018963), *S. mansoni* SmAChE1, (AAQ14321.1*), S. haematobium* ShAChE1, (AAQ14322.1), *S. bovis* SbAChE1, (AAQ14323.1), *S. japonicum* SjAChE1, (ANH56887.1), *Echinococcus granulosus* EgAChE, (EUB57267.1) *Echinococcus multilocularis* EmAChE, (CDS43027.1), *Clonorchis sinensis* CsAChE, (GAA53463.1), *Opisthorchis viverrini* OvAChE, (XP_009168237.1), *Caenorhabditis briggsae* CbAChE, (XP_002643631.1), *Caenorhabditis elegans* CeAChE, (NP_510660.1), *Trichuris trichiura* TtAChE, (CDW55373.1), *Trichinella spiralis* TsAChE, (XP_003374589.1), *Strongyloides ratti* SrAChE, (CEF63281.1), *Homo sapiens* HsAChE, (NP_000656) and the electric eel *Torpedo californica* TcAChE, (P04058.2). Numbers on the branches reflect the branch support value, and the scale bar indicates 30% amino acid sequence divergence.

**Figure 2A** shows that treating adult male worms with siRNAs targeting SmAChE1 results in the successful suppression of this gene (by >75%, p<0.01 v either control). Three different siRNAs were evaluated, and preliminary analysis showed that all 3 siRNAs provided similar levels of gene suppression; therefore, SmAC1-siRNA1 was chosen in all subsequent RNAi experiments (**Table S1**). **Figure 2B** shows that this suppression had no significant impact on the ability of live worms to cleave exogenous acetylthiocholine (**Figure 2B**). There was no significant difference in the AChE activity displayed by the SmAChE1gene-suppressed worms versus controls. This result strongly suggests that a different gene, encoding a surface AChE, is responsible for the live worm activity detected. Therefore, as described in Methods, we performed an extensive *in silico* analysis of schistosome DNA databases in order to identify other potential acetylcholinesterase genes encoding proteins that might be responsible. In this manner Smp_136690 was identified to encode a strong candidate gene that we now designate SmTAChE (https://parasite.wormbase.org/Schistosoma_mansoni_prjea36577/Info/Index/; database version WBPS17). This gene is 23,068 bp (from the start to the stop codon) and, as depicted in **Figure 3A** (top), consists of 4 exons and 3 introns, unlike the SmAChE1 gene (Smp_154600) which contains 9 exons (**Figure 3A**, bottom). All introns have the consensus splicing site sequence (5’-GT/AG-3’). Both AChE genes are found on chromosome number 1, the largest autosomal chromosome; they are ~300 kb apart. Several oligonucleotides were designed based on the SmTAChE genomic sequence and used to amplify the complete 2,085 bp open reading frame, using adult *S. mansoni* cDNA as template. The GenBank accession number for the complete nucleotide sequence of the SmTAChE cDNA is OP018961. The ORF codes for the SmTAChE polypeptide of 694 amino acids. This is essentially a newly annotated version of a previously reported shorter (489 amino acid) sequence that was designated SmAChE2 (30). Due to substantial differences between the predicted sequences of these proteins (as shown in **Figure S1**), we elect to use the SmTAChE name for the new sequence reported here.

As shown in **Figure 3B**, phylogenetic analysis shows that SmTAChE (red text) is closely related to homologs from *S. haematobium* and *S. japonicum* (described below) and these are quite distinct from previously described schistosome AChE proteins (SmAChE1 and its homologs). All trematode AChEs (from schistosomes, liver flukes and cestodes) form a distinct clade, distant from the AChEs of animals from other phyla, such as nematodes and vertebrates. The multiple sequence alignment of all the sequences used to generate the phylogenetic tree is presented in **Figure S2**.

**Figure 4:**
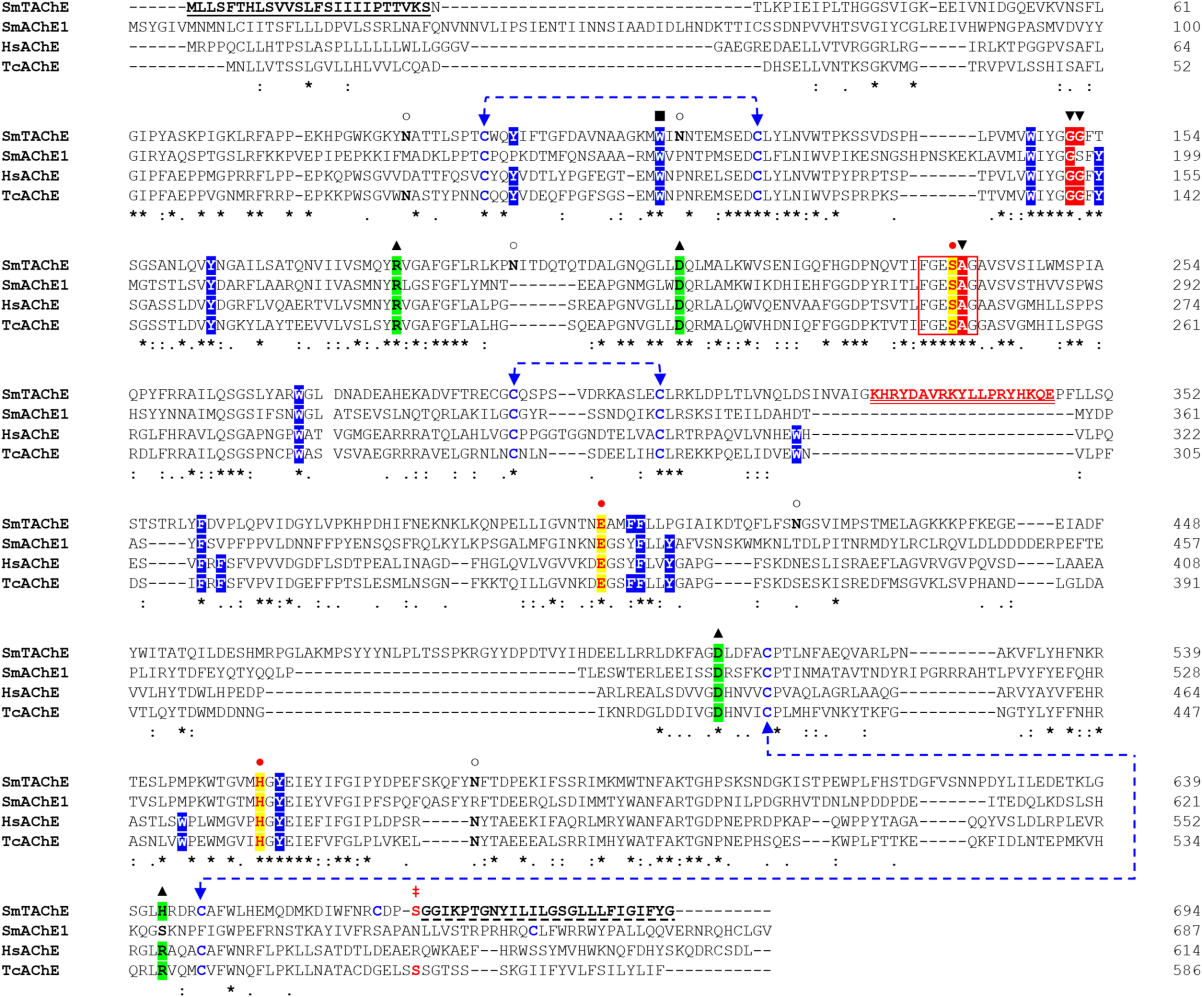
Alignment of the amino acid sequences of selected AChE proteins. The amino acid sequence of SmTAChE (Accession number OP018961), SmAChE1 (AAQ14321.1), human AChE (HsAChE, GPI-modified H form; NP_000656) and the Electric eel, *Torpedo californica* (TcAChE*;* GPI-modified form, P04058.2) were aligned using Clustal W. The catalytic triad residues (S^239^, E^401^, H^553^, where amino acid positions correspond to the numbering of the SmTAChE sequence) are in red and indicated with 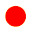 and shaded in yellow. The 6 amino acid signature sequence (^236^FGESAG^241^) of cholinesterases around the active serine residue (S^239^) is boxed. The choline-binding site (W^115^) is indicated with ▪. The three intrachain disulfide bonds formed using six conserved cysteines (C^97^-C^125^, C^293^-C^306^, C^514^-C^647^) are indicated with dashed blue lines. The four conserved charged residues involved in forming two salt-bridges are marked with ▴ and shaded in green. The positions of the 14 aromatic residues that line the catalytic gorge in *T. californica* AChE are shaded in blue (9 are identical in SmTAChE). The oxyanion hole residues are indicated with ▾ and shaded in red. Five potential N-glycosylation sites (with the consensus sequence NXS/T) are marked with **○**. The amino acid sequence of the unique peptide (K^329^-E^346^) used to generate antibodies for SmTAChE is in red and double underlined. The signal peptide of SmTAChE (M^1^-S^25^) is underlined. The GPI anchor sequence of SmTAChE (G^669^ - G^694^) is indicated with a dashed underline, and the omega (ω)-residue (S^668^) is indicated with 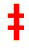. Identical residues in all sequences are marked with an asterisk (*); strongly similar residues (conserved substitution) are indicated with a colon (): weakly similar residues (semi-conserved) indicated with a dot (.), and gaps, introduced to allow maximum alignment, are marked with dashes (–). The numbers (right) tally the amino acid count per line for each sequence.

The SmTAChE sequence exhibits 36% amino acid sequence identity with SmAChE1, and 35% identity with the human AChE enzyme (HsAChE). As illustrated in **Figure 4**, SmTAChE contains all amino acids considered essential for AChE activity: the catalytic triad (S^239^, E^401^, H^553^), the choline-binding site (W^115^), six cysteines responsible for 3 intrachain disulfide bonding, the four charged residues involved in forming two salt-bridges, nine amino acids out of 14 aromatic residues that line the catalytic gorge in *Torpedo californica* AChE. In addition, SmTAChE contains the essential residues that form the oxyanion hole (G^151^, G^152^, and A^240^) which plays an important role in catalysis (45). The SmTAChE sequence is predicted to contain three intrachain disulfide bonds formed between six conserved cysteine residues (C^97^-C^125^, C^293^-C^306^, C^514^-C^647^, indicated by blue dashed arrows in **Figure 4**) which are considered essential for correct secondary structure formation. Importantly, SmTAChE also contains a predicted signal peptide (M^1^ - S^25^) and a strong predicted C-terminal GPI anchor sequence (^669^GGIKPTGNYILILGSGLLLFIGIFYG^694^, dashed underline in **Figure 4**) (30). The GPI-anchoring site (omega (ω)-residue, S^668^) is indicated by **‡** in **Figure 4**.

### SmTAChE developmental expression

**Figure 5:**
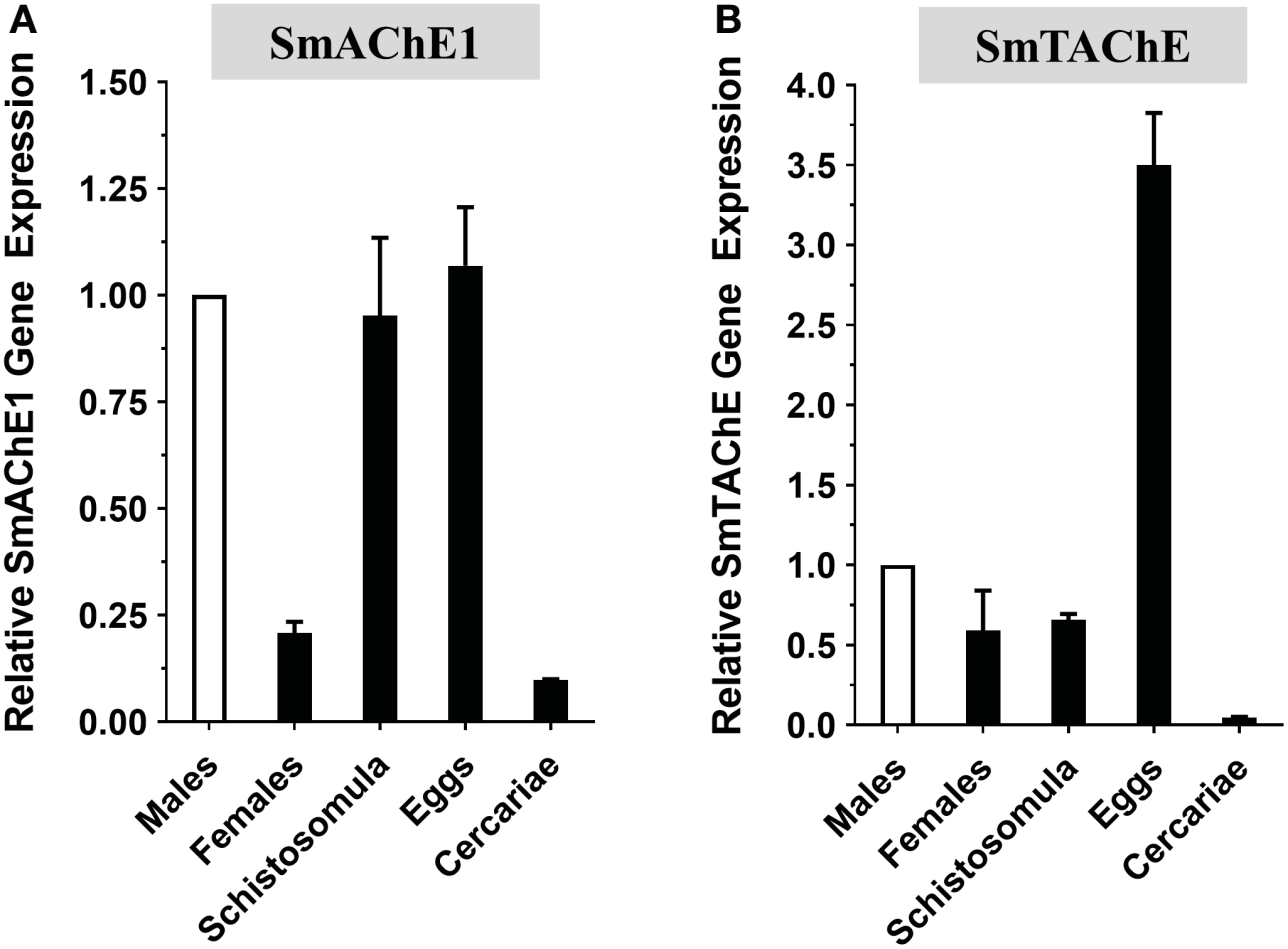
Developmental expression of acetylcholinesterase genes in different *S. mansoni* life stages as determined by RT-qPCR. **(A)** SmAChE1 gene expression. **(B)** SmTAChE Gene expression. All values are relative to the gene expression level of male parasites, set at 1 (Mean ± SD, n=3).

The developmental expression of both the SmAChE1 and SmTAChE genes were examined in several schistosome life stages by RT-qPCR. As shown in **Figure 5**, both genes have lowest relative expression in the cercarial stage and, in both cases, expression increases greatly following infection – in schistosomula and in adult males and females. Both genes are very well expressed in eggs, strikingly so for SmTAChE. Based on available transcriptomic datasets (https://v7test.schisto.xyz/), the expression of both genes is high in adult male parasites.

### Immunolocalization of SmTAChE

**Figure 6:**
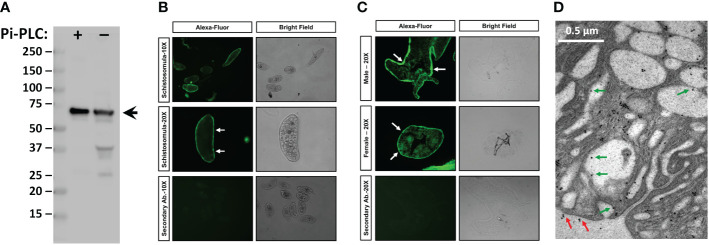
Immunolocalization of SmTAChE. **(A,** left) Anti-SmTAChE antibodies bind to a protein of the expected size of SmTAChE (~75 kDa, arrow) as seen by western blot analysis in a protein preparation obtained following Pi-PLC treatment of live adult males (**+**) and in a total male lysate (not treated with Pi-PLC, **-**). Numbers to the left indicate molecular mass markers (kDa). **(B)** Immunostaining of SmTAChE in 7-day old cultured schistosomula. **(C)** Immunostaining of SmTAChE in sections of adult male longitudinal section; (**C**, top panel) and adult female (cross section; **C**, middle panel) parasites. Magnification level (10X or 20X) is indicated to the left. An Alexa-Fluor 488 conjugated secondary antibody was used for detection and was used as a control. Both fluor (left panels) and bright (right panels) fields are shown for each section. Strong tegumental staining is seen in schistosomula and adults (white arrows). **(D)** Electron micrograph of the adult tegument showing immunogold labeling of SmTAChE. Red arrows indicate localization of gold particles on the external surface of the worm and green arrows indicate the presence of some gold particles lining tegumental pits.

To localize SmTAChE, we first generated an anti-SmTAChE antibody targeting a unique synthetic peptide (K^329^-E^346^, underlined red text in **Figure 4**) found only in SmTAChE, and not in SmAChE1. As shown in **Figure 6A**, the antibody detects a major protein band of the expected size of SmTAChE (~75kDa, arrow) in extracts of adult male parasites (arrow), both among the Pi-PLC released proteins from live male parasites (**Figure 6A**, +Pi-PLC) and in a total male worm lysate (-Pi-PLC). Immunolocalization of SmTAChE was carried out using the anti-SmTAChE antibodies and whole, fixed 7-day cultured schistosomula (**Figure 6B**) or fixed, frozen sections of adult parasites (**Figure 6C**). Strong SmTAChE staining is observed, very predominantly in the tegument; a clear “green ring” of peripheral staining around the schistosomula is revealed (**Figure 6B**, white arrows). Likewise in adult worms, clear tegumental staining is revealed in both the male longitudinal section (**Figure 6C**, top white arrows) and in the female cross section (**Figure 6C**, middle panel, white arrows). Control parasites, exposed to secondary antibody alone, do not display signal in the tegument or elsewhere, in either adult parasites or schistosomula (**Figure 6B, C**, bottom panels). Further analysis using e.g., confocal microscopy would help confirm expression of SmTAChE in the tegument of schistosomula. Localization of SmTAChE by immunogold electron microscopy (**Figure 6D**) does confirm that the protein is distributed in adult worm tegumental membranes. Immunogold particles are seen scattered widely throughout the section, sometimes in small clusters, including at the host interactive surface (red arrows). In addition, immunogold particles are also seen lining tegumental pits (green arrows). Very few immunogold particles are seen in control sections incubated with gold-labeled secondary anti-rabbit antibody alone (**Figure S3**).

### The SmTAChE gene encodes the surface AChE

**Figure 7:**
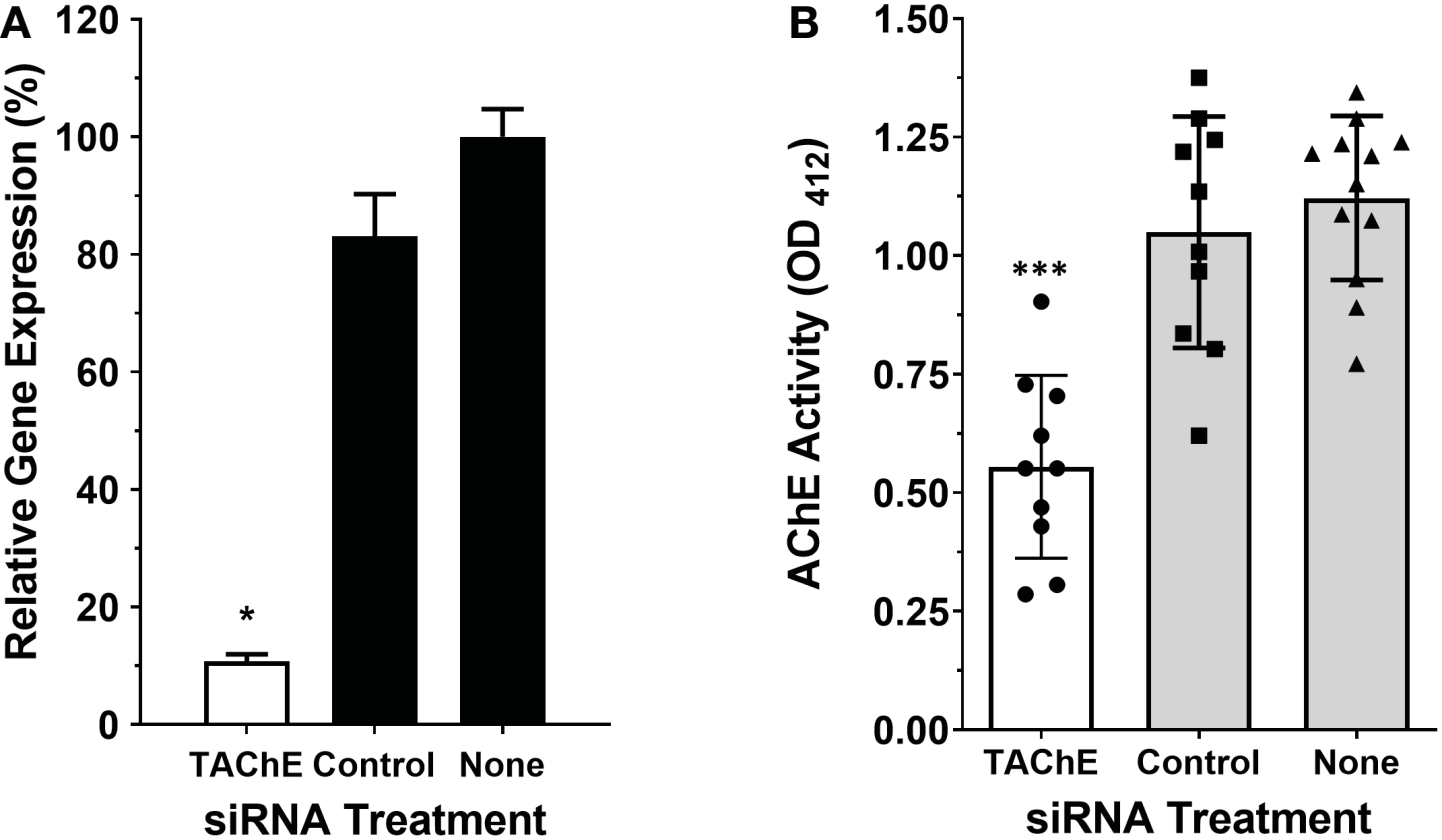
The SmTAChE gene encodes the surface AChE. Adult male parasites were treated either with an siRNA targeting SmTAChE, or with an irrelevant siRNA (Control) or with no siRNA (None), as indicated. **(A)** Treating parasites with siRNA targeting SmTAChE significantly suppressed the expression of this gene (by >90%; white bar, *p < 0.05 ANOVA) compared to controls, as revealed by RT-qPCR. **(B)** The AChE activity of all three groups of live parasites, one week after treatment, is shown, where each point represents the data from an individual parasite, and the bars indicates the mean for the group ( ± SD). Mean surface AChE activity is significantly lower in parasites treated with siRNA specific for SmTAChE (white bar) compared to parasites treated with control siRNA or with no siRNA (None) (grey bars, ***p < 0.0001, ANOVA, n ≥10).

To evaluate whether the SmTAChE gene codes for the enzyme responsible for surface AChE activity, we first knocked-down expression of the gene using RNAi using 2 different siRNAs. Both siRNAs gave similar levels of suppression, and for all subsequent experiments SmAC2-siRNA1 was used. **Figure 7A** shows ~90% SmTAChE gene suppression in parasites treated with an siRNA targeting the gene compared to control parasites treated with a control (irrelevant) siRNA or no siRNA (none), as determined by RT-qPCR. To assess the impact of the knock-down on the surface AChE, the ability of live worms to cleave exogenous acetylthiocholine was compared between the groups, 7 days after RNAi treatment. As shown in **Figure 7B**, mean surface AChE activity is significantly reduced in live male parasites treated with siRNA specific for the SmTAChE gene, versus controls (p < 0.0001). No visible changes were observed to SmTAChE gene-suppressed parasites maintained in culture for 14 days, as assessed by light microscopy.

### SmTAChE is essential for parasite survival *in vivo*

**Figure 8:**
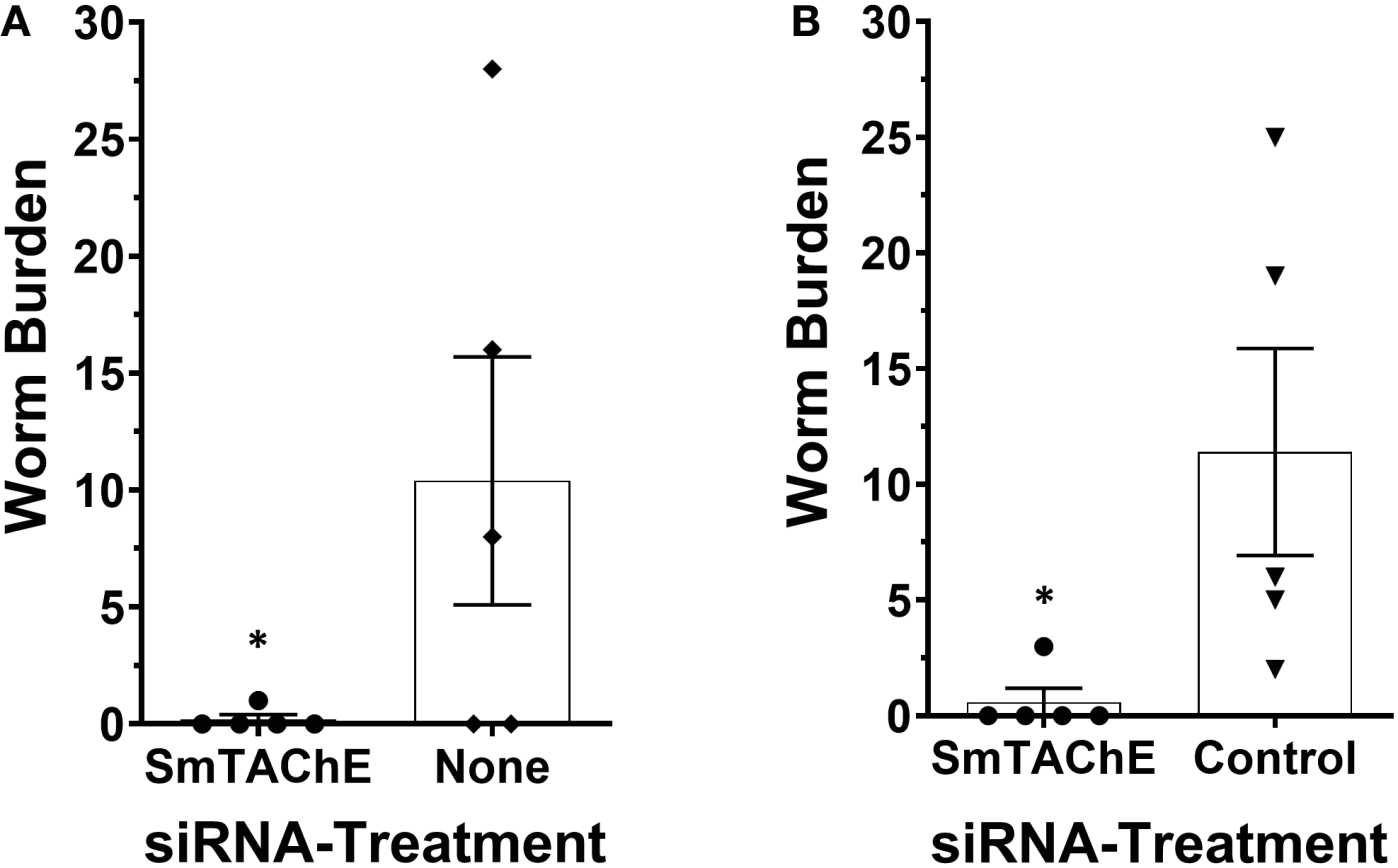
SmTAChE is essential for parasite survival *in vivo*. Schistosome recovery from mice 6 weeks after infection with SmTAChE-suppressed or control parasites. Results from two independent experiments are shown. **(A)** schistosomula were treated with siRNA specific for SmTAChE, or with no siRNA, prior to infection and in **(B)** schistosomula were treated with siRNA specific for SmTAChE or with control siRNA prior to infection. Each point represents the worm burden from one mouse, and the line represents the mean ( ± SD) for the group. In both instances, treatment with SmTAChE siRNA significantly reduced the number of recovered parasites (*P < 0.04, Students’ t test).

To investigate whether SmTAChE has an impact on parasite survival *in vivo*, we first suppressed its expression in schistosomula and, after 4 days in culture, these parasites were used to infect mice. Control mice were infected either with schistosomula treated with an irrelevant (control) siRNA or no siRNA, as previously described (12, 44). Mice were perfused six weeks later, and worm burdens were analyzed. As shown in **Figure 8**, in two independent experiments, almost no parasites were recovered from mice infected with schistosomula whose SmTAChE gene was suppressed compared to mice infected with schistosomula treated with no siRNA (**Figure 8A**) or mice infected with schistosomula treated with control siRNA (**Figure 8B**) (p < 0.05, for both experiments, Student’s t test). This important result shows that SmTAChE performs a vital function that is key for parasite survival.

### Identification of SmTAChE homologs in *S. haematobium* and *S. japonicum*

Analysis of the *S. haematobium* genome resulted in the identification of a gene (MS3_0012973) encoding a potential protein with 85% sequence identity to SmTAChE, which we designate ShTAChE. The complete nucleotide sequence of ShTAChE cDNA is deposited at the NCBI GenBank under the accession number OP018962. In the case of S*. japonicum*, no single contig or scaffold was identified that contained a complete SmTAChE homolog. However, as detailed in Methods, two different contigs were identified in WormBase ParaSite, each encoding part of an SmTAChE homolog; based on these sequences and using the RACE technique, we isolated the complete *S. japonicum* TAChE coding region. The complete nucleotide sequence of SjTAChE cDNA is deposited at NCBI GenBank under the accession number OP018963. The predicted SjTAChE protein has 71% sequence identity with SmTAChE and 69% identity with ShTAChE. Analysis of the gene structures of the three schistosome TAChE genes shows them to be very similar; as shown in **Figure S4**, all genes contain 4 exons and 3 introns (**Figure S4**) and with all exon-splicing sites being identical in the three genes. **Figure S5** shows the predicted amino acid sequences of the three schistosome TAChE proteins. Similar to SmTAChE, both ShTAChE and SjTAChE proteins are predicted to have a signal peptide and a GPI anchoring signal with conserved ω-site, as well as all motifs conserved in other members of the AChE family.

### Surface AChE activity of the three major human schistosome species

**Figure 9:**
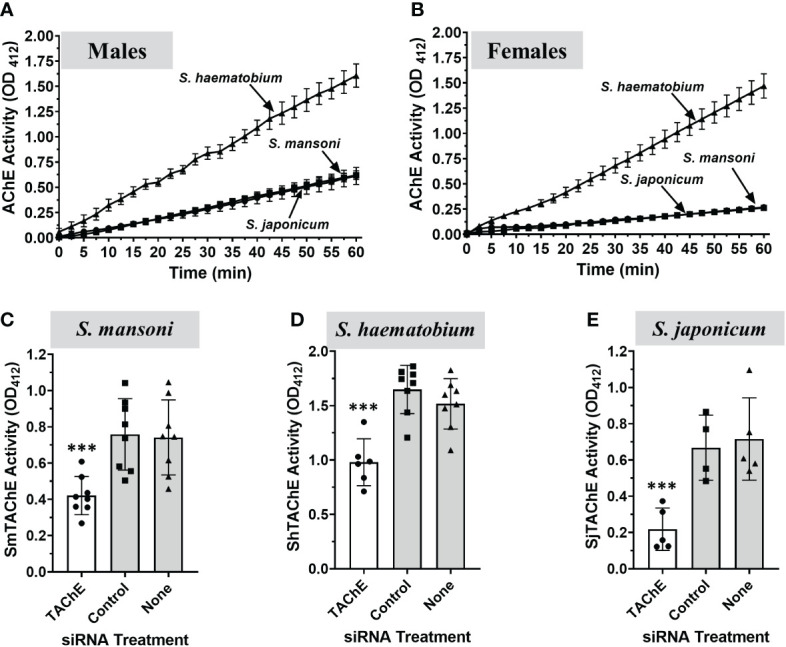
Surface AChE activity (mean OD_412_ ± SD) in live individual adults of the three medically most important schistosome species *S. mansoni*, *S. haematobium* and *S. japonicum*, as indicated (n≥5). **(A)** Live individual adult male parasites. **(B)** Live individual adult female parasites. *S. haematobium* parasites (both male and female) exhibit significantly higher surface AChE activity versus *S. mansoni* or *S. japonicum* parasites (p < 0.001, Two-Way ANOVA). No significant difference is observed between live *S. mansoni* and *S. japonicum* adult parasite AChE activity. **(C–E)** A universal siRNA targeting the tegumental AChE gene of the three species was designed and used to knock-down the expression of this gene in adult male parasites. Seven days later the surface AChE activity (mean OD_412_ ± SD) was measured in live, gene-suppressed versus control *S. mansoni* **(C)**, *S. haematobium* **(D)** or *S. japonicum* **(E)** adult males. Each point represents the data from one worm, and the line represents the mean ( ± SD) for the group. In all cases, gene suppression resulted is significant reductions in surface AChE activity (**C**, *S. mansoni;* ***p < 0.001), (**D**, *S. haematobium*; ***p < 0.0001) and (**E**, *S. japonicum*; ***p < 0.008, all by ANOVA). Values obtained at 60 min are presented.

Here we compare the surface AChE activity displayed by male and female parasites of the three major human schistosome species. As shown in **Figure 9**, individual live adult male (**Figure 9A**) and live adult female (**Figure 9B**) schistosomes from all three species cleave exogenous acetylthiocholine. *S. haematobium* parasites have the highest AChE activity; individual male *S. haematobium* have approximately twice the surface activity found in individual *S. mansoni* or *S. japonicum* parasites, while individual female *S. haematobium* parasites have almost 7 times the surface activity of individual females of the other two schistosome species. (Quantitative data reported in **Table S3**) It is noteworthy that individual female *S. haematobium* display similar surface AChE activity to individual male parasites (**Table S3**). For *S. mansoni* and *S. japonicum*, live individual male AChE activity is about twice that of individual females.

The TAChE genes of all three schistosome species were targeted for suppression by treating male worms with a universal siRNA that targets the TAChEs of all three. In each case this treatment resulted in significant reductions in surface AChE activity seven days post treatment (white bars) compared to control parasites treated either with control siRNA or with no siRNA (grey bars) (**Figures 9C-E**).

## Discussion

We demonstrated here for the first time that live, intact, intravascular-stage schistosomes can cleave exogenous acetylthiocholine. Adult male and female *S. mansoni*, as well as schistosomula, display this activity, as do freshly isolated parasite eggs. All such activity is blocked in the presence of the selective AChE inhibitor BW284c51. The majority of total adult male worm AChE activity (~70%) can be attributed to the action of the surface AChE. While a tegumental butyrylcholinesterase (BuChE) has been identified in *S. mansoni* (SmBuChE (30),), we find that intact, live parasites display no evidence of host-interactive BuChE activity, suggesting that SmBuChE is not expressed on the external surface of the intravascular-stage worms. In addition, we were unable to detect secreted AChE activity, showing that AChE remains attached to the parasites surface and is not excreted or secreted by the worms to any great extent. In agreement with this, previous work detected AChE activity only in medium where live parasites had been treated with Pi-PLC, but not in the medium of control parasites (46).

A gene encoding an AChE was previously identified in *S. mansoni*, *S. haematobium*, *S. bovis* and *S. japonicum* (17, 21, 28, 29). The worms had been reported to display AChE activity both internally as well as on their external tegumental membranes and it was originally proposed that the identified gene encoded both internal and tegumental enzymes (17). However, we show here that suppression of this gene in *S. mansoni* (SmAChE1) using RNAi failed to significantly impact the surface AChE activity of live adult male parasites. In addition, neither SmAChE1 (and its homologs in other schistosome species), nor a second AChE identified in *S. mansoni* (SmAChE2 (30),), were predicted to contain a consensus C-terminal GPI anchoring sequence. Therefore, we hypothesized that another gene coded for the surface AChE. *In silico* analysis of the *S. mansoni* genome revealed the presence of a gene potentially encoding a 694 amino acid protein that has a high degree of identity with the previously published 488 amino acid SmAChE2 (30). Despite the fact that both sequences are encoded by the same gene (Smp_136690), substantial differences have emerged between the published SmAChE2 protein (30) and the sequence we present here. Our predicted AChE is essentially a newly annotated version of the previous sequence, and we elect to use the name SmTAChE for this enzyme. We cloned the complete coding sequence of this gene and, as detailed in Results, identified all domains and essential amino acids required for AChE activity in its predicted amino acid sequence. We have sequenced and analyzed multiple PCR products generated using adult parasite cDNA as template, but have never found a sequence that matches the published SmAChE2 sequence (30). Importantly, our SmTAChE sequence contains a leader sequence and a consensus GPI anchoring domain, supporting the biochemical evidence that the tegumental AChE is GPI linked (21, 27, 42). Indeed, we show here that experimental treatment of live schistosomes with Pi-PLC releases SmTAChE. Interestingly, it has been previously demonstrated that treating parasites with Pi-PLC significantly upregulates the synthesis of AChE (47).

Immunolocalization experiments reported here, using antibodies that are specific for SmTAChE, showed that the protein is prominently expressed in the tegument of the schistosomula and adult parasite stages, as expected if this protein is responsible for surface AChE activity. Most importantly, knocking down the expression of the SmTAChE gene resulted in a significant reduction in the surface AChE activity displayed by live parasites. These data prove that the tegumental surface AChE of *S. mansoni* is encoded by the SmTAChE gene and help to clarify longstanding confusion regarding schistosome AChE activity. To our surprise, SmTAChE expression is not seen to be especially enriched in tegumental cells (neither cluster 1 nor cluster 2) in an *S. mansoni* single cell dataset (48, 49). Instead, expression is detected in many cell types with enrichment noted in several distinct neuronal cell groups and in flame cells (48, 49). More work is needed to better reconcile the transcriptomic versus tegument proteomic data sets.

Presumably SmTAChE is the protein earlier characterized as having a sedimentation coefficient of 6.5S that does not bind to heparin but that can be released from intact parasites by Pi-PLC treatment (26, 27). In support of this and as noted earlier, treatment of live schistosomes with Pi-PLC releases SmTAChE. We infer that SmAChE1 is the internal protein earlier characterized as having a sedimentation coefficient of 8S that binds heparin (26). Genes encoding both proteins are expressed in all intra-mammalian life stages of the parasites, and both exhibit minimal relative expression in the cercarial life stage.

In other animals, a key function of the AChE enzyme is to terminate synaptic transmission at cholinergic synapses by hydrolyzing the neurotransmitter acetylcholine, and we hypothesize that SmAChE1 largely fulfills this function in schistosomes. However, the function of the non-neuronal, tegumental enzyme SmTAChE is unknown (50). To examine the importance of this tegumental AChE enzyme for *S. mansoni in vivo*, SmTAChE gene-suppressed versus control schistosomula were used to infect mice and, after 6 weeks, worm burdens were compared between the groups. Almost no parasites are recovered from mice infected with schistosomula whose SmTAChE gene was suppressed compared to controls. This important result shows that the function(s) performed by SmTAChE are vital for parasite survival. The essential function fulfilled by SmTAChE is unclear. The enzyme may influence host vascular physiology, given that acetylcholine is a vasodilator (51–53). Additionally, SmTAChE may have an immunoregulatory role since several immune cells respond to acetylcholine (54, 55) and, due to SmTAChE action, it seems likely that immune signaling *via* acetylcholine would be impaired, at least in the vicinity of the worms. Glucose uptake is reported to be enhanced in the presence of acetylcholine in adult *S. haematobium* and *S. bovis* but not *S. mansoni* (56) and it is possible that the action of tegumental AChEs could impact this. Beyond schistosomes, AChEs are now reported to be distributed in a variety of non-neuronal tissues including in hematopoietic, osteogenic and various neoplastic cells, where the enzymes are predicted to express non-classical (and possibly non-enzymatic) activities (21, 57–59). Currently, there is substantial effort to understand the physiological significance and the molecular mechanisms of these non-neuronal cholinergic activities (60–65).

Since the suppression of SmTAChE gene expression severely impaired the ability of the worms to establish a robust infection, this validates SmTAChE as a therapeutic target for schistosomiasis. Indeed, it has been shown that human schistosomiasis can be treated using the drug metrifonate, an organophosphorus compound (13, 66). The active metabolite of metrifonate, dichlorvos (2,2-dichlorovinyl dimethyl phosphate), acts by inhibiting AChEs. It was suggested that tegumental AChE is the target for this therapy (20). While metrifonate is no longer commercially available as an anti-schistosome agent due to the need for multiple doses, reduced efficacy compared to the newer drug praziquantel (PZQ), and higher specificity to human AChE which can result in toxicity (66, 67), its earlier use in humans highlights the potential value of identifying schistosome-specific AChE inhibitors (against SmTAChE and/or SmAChE1) as novel therapies.

Since any new therapy for schistosomiasis should ideally target all three medically important species, we looked for evidence of surface AChE activity in *S. japonicum* and *S. haematobium*, in addition to *S. mansoni*. Adult male and female worms of all three species were observed to cleave exogenous acetylthiocholine with *S. haematobium* parasites exhibiting the highest surface AChE activity, when compared to the other two species. Both *S. mansoni* and *S. japonicum* adult worms express very similar levels of surface AChE activity. In contrast, individual adult male *S. haematobium* parasites display about two-fold greater activity compared to individual males of either *S. japonicum* or *S. mansoni*. Additionally, female *S. haematobium* exhibits strikingly high relative AChE surface activity - almost seven times that found in the other two species. These data are in general agreement with reports that tegument extracts of adult *S. haematobium* display substantially higher AChE activity compared to equivalent tegument extracts of *S. mansoni* (20). The requirement for such high surface AChE activity by *S. haematobium*, and whether it is related to living in its distinct, preferred intravascular niche (the vesicle plexus), is not known. Similarly, why *S. mansoni* eggs should display such a very high relative SmTAChE expression level (>3 times that of adult males) is unclear.

Finally, we investigated whether the surface AChEs of *S. haematobium* and *S. japonicum* are encoded by SmTAChE homologs. We first identified and cloned clear homologs from *S. haematobium* (ShTAChE) and *S. japonicum* (SjTAChE) and we identified that the genes encoding these proteins possess a very similar structure across all three species, and all three proteins are predicted to possess signal peptides and GPI anchoring sites. We next targeted the surface AChE gene from each species for suppression by RNAi. This yielded parasites with a significantly diminished ability to cleave exogenous acetylthiocholine compared to controls, confirming that the identified genes indeed encode the tegumental AChE in the three schistosome species.

The two *S. mansoni* AChE gene structures are quite divergent; SmAChE1 has 9 exons while SmTAChE has 4. In addition, the 687 amino acid SmAChE1 and the 694 amino acid SmTAChE display just moderate (~35%) amino acid sequence identity and this is about the same as that displayed by either of the schistosome AChEs versus (evolutionarily distant) human AChE (614 amino acids). Given the moderate level of amino acid sequence identity between the schistosome AChEs versus the human enzyme, it is likely that drugs preferentially inhibiting the schistosome enzymes could be developed. Unlike schistosomes, the human genome encodes a single AChE, and this makes any differential inhibitor screening (i.e., testing for inhibition of schistosome *vs*. human AChE) very feasible.

Heterologous anti-AChE antibodies (raised by immunizing rabbits with AChE from an electric eel, *Eletrophorus electricus*) bind to live *S. mansoni* schistosomula (68). Incubating these parasites with guinea pig serum (as a source of complement) results in ~ 50% killing of the parasites (68). Similarly, incubating schistosomula with antibodies raised against *S. mansoni* cercarial AChE (in the presence of guinea pig serum) leads to ~80% parasite killing (69). We infer that in these experiments the anti-AChE antibodies drive this effect by binding to the SmTAChE that is exposed on the external surface of the worms; this provides a strong rationale for also considering surface located SmTAChE as a novel anti schistosome vaccine candidate. Blocking SmTAChE function using either new drugs, or immunologically, offers a new therapeutic approach to control schistosomiasis.

## Data availability statement

The datasets presented in this study can be found in online repositories. The names of the repository/repositories and accession number(s) can be found in the article/**Supplementary Material**.

## Ethics statement

The animal study was reviewed and approved by The Institutional Animal Care and Use Committee (IACUC) of Tufts University and all animal work was done in the vivarium at Cummings School of Veterinary Medicine, Tufts University.

## Author contributions

PS: Conceptualization, data curation, writing and editing the manuscript. AD: Conceptualization, data curation and analysis. performed the experiments, writing the first draft and finalizing the manuscript. All authors contributed to the article and approved the submitted version.

## Supplementary material

Supplementary Figure 1 Sequence comparison of SmTAChE and SmAChE2. Both sequences derive from the gene Smp_136690; however, the SmTAChE protein (accession number OP018961) is substantially different from the published SmAChE2 sequence (30). The SmAChE2 sequence lacks a signal peptide(M^1^-S^25^) underlined in the SmTAChE sequence) and a GPI-anchoring signal (G^669^-G^694^ double underlined in SmTAChE) and, likely due to differences in annotation, is missing many blocks of amino acids compared to SmTAChE. Differences between the sequences are depicted by red text highlighted in yellow.

Supplementary Figure 2 Alignment of multiple AChE sequences using Muscle (http://www.phylogeny.fr/). These data were used to generate the phylogenetic tree shown in . All accession numbers are indicated in the legend. Similar residues are highlighted in blue as most conserved according to BLOSUM62. Average BLOSUM62 score: Max: 3.0, Mid: 1.5, Low: 0.5.

Supplementary Figure 3 Control electron micrograph (EM) image. Very few immunogold particles are seen in sections, such as that shown, that were incubated with gold-labeled secondary anti-rabbit antibody alone.

Supplementary Figure 4 A schematic representation of the conserved tegumental AChE gene structure of three schistosome species. The gene encoding the tegumental AChE of *S. mansoni* (SmTAChE, topo) is compared to that of *S. haematobium* (ShTAChE, middle) and *S. japonicum* (SjTAChE, bottom). The sizes of exons and introns are indicated (numbers represent base pairs). The//indicates that the complete intron 1 of SjTAChE has not yet been characterized.

Supplementary Figure 5 Alignment of the amino acid sequences of the tegumental acetylcholinesterases (AChEs) of three schistosome species. The amino acid sequence of the tegumental *Schistosoma mansoni* AChE (*Sm*TAChE, accession number OP018961), tegumental *S. haematobium* AChE (*Sh*TAChE, accession number OP018962) and tegumental *S. japonicum* AChE (*Sj*TAChE, accession number OP018963) were aligned using Clustal W. The catalytic triad residues (S^239^, E^401^, H^553^, where amino acid positions correspond to the numbering of the *Sm*TAChE sequence) are in red and indicated with **●**. The signature sequence (^236^FGESAG^241^) of cholinesterases around the active serine residue (S^239^) is boxed. The choline-binding site (W^115^) is indicated with ▪. The three intrachain disulfide bonds formed using six conserved cysteines are indicated with dashed blue lines. The boxed cysteine residue (C^665^) is predicted to be involved in dimer formation. Predicted signal peptides are underlined and predicted GPI anchoring motifs are indicated with a dashed underline. All three sequences have a conserved omega (ω)-site (indicated with ‡). All other domains are described in the legend to . Red arrows (↓) depict amino acids encoded by DNA sequences at exon/exon junctions, further highlighting their conservation.
