# Supplementary material for: A novel, non-neuronal acetylcholinesterase of schistosome parasites is essential for definitive host infection
Source: Front Immunol. 2023 Jan 31;14:1056469. doi: 10.3389/fimmu.2023.1056469 (PMC9927205; doi:10.3389/fimmu.2023.1056469)
Supplement: Supplementary Figure 1 — Sequence comparison of SmTAChE and SmAChE2. Both sequences derive from the gene Smp_136690; however, the SmTAChE protein (accession number OP018961) is substantially different from the published SmAChE2 sequence (30). The SmAChE2 sequence lacks a signal peptide(M1-S25) underlined in the SmTAChE sequence) and a GPI-anchoring signal (G669-G694 double underlined in SmTAChE) and, likely due to differences in annotation, is missing many blocks of amino acids compared to SmTAChE. Differences between the sequences are depicted by red text highlighted in yellow. [file DataSheet_1.pdf]

```
SmTAChE   MLLSFTHLSVVSLFSIIIIPTTVKSNTLKPIEIPLTHGGSVIGKEEIVNIDGQEVKVNSF   60
SmAChE2   ---------------------------------MLTHGGSVIGKEEIVD----GVKVNSF   23

SmTAChE   LGIPYASKPIGKLRFAPPEKHPGWKGKYNATTLSPTCWQYIFTGFDAVNAAGKMWINNTE   120
SmAChE2   LGIPYASKPIGKLRFAPPEKHPGWKGKYNATTLSPTCWQYIFTGFDAV-AAGKMWINNTE   82

SmTAChE   MSEDCLYLNVWTPKSSVDSPHLPVMVWIYGGGFTSGSANLQVYNGAILSATQNVIIVSMQ   180
SmAChE2   MSEDCLYLNVWTPKS----SHLPVMVWIYGGGFTSGSANLQVYNGAILSATQNVIIVSMQ   138

SmTAChE   YRVGAFGFLRLKPNITDQTQTDALGNQGLLDQLMALKWVSENIGQFHGDPNQVTIFGESA   240
SmAChE2   YRVGAFGFLRLK-------QTDALGNQGLLDQLMALKWVSENIGQFHGDPNQVTIFGESA   191

SmTAChE   GAVSVSILWMSPIAQPYFRRAILQSGSLYARWGLDNADEAHEKADVFTRECGCQSPSVDR   300
SmAChE2   GAVSVSILWMSPIAQPYFRRAILQSGSLYARWGLDNADEAHEKADVFTRECGCQSA----   247

SmTAChE   KASLECLRKLDPLTLVNQLDSINVAIGKHRYDAVRKYLLPRYHKQEPFLLSQSTSTRLYF   360
SmAChE2   --SLECLRKLDPLTLVNQLDSL---------------------------------TRLYF   272

SmTAChE   DVPLQPVIDGYLVPKHPDHIFNEKNKLKQNPELLIGVNTNEAMFFLLPGIAIKDTQFLFS   420
SmAChE2   DVPLQPVIDG---------------------------------------IAIKDTQFLFS   293

SmTAChE   NGSVIMPSTMELAGKKKPFKEGEEIADFYWITATQILDESHMRPGLAKMPSYYYNLPLTS   480
SmAChE2   NGSVI---TMELAGKKKPD--GEEIASYY-------------------------------   317

SmTAChE   SPKRGYYDPDTVYIHDEELLRRLDKFAGDLDFACPTLNFAEQVARLPNAKVFLYHFNKRT   540
SmAChE2   ---------------YEELLRRLDKFAGDLDFACPTLNFAEQVAR-PNAKVFLYHFNKRT   361

SmTAChE   ESLPMPKWTGVMHGYEIEYIFGIPYDPEFSKQFYNFTDPEKIFSSRIMKMWTNFAKTGHP   600
SmAChE2   ESLPMPKWTGVMHGYEIEYIFGIPYDPE----FYNFTDPEKIFSSRIMKMWTNFAKTGHP   417

SmTAChE   SKSNDGKISTPEWPLFHSTDGFVSNNPDYLILEDETKLGSGLHRDRCAFWLHEMQDMKDI   660
SmAChE2   SKSND------EWPLFHST------DPDYLILEDETKLGSGLHRDRCAFWLHEMQDMKDI   465

SmTAChE   WFNRCDPSGGIKPTGNYILILGSGLLLFIGIFYG                           694
SmAChE2   W---CDPSLRLYFISLILLHSRDSSVL-------                           489
```

Figure S1

Figure S2

```
SjAChE1   ------mNMiyiainyyIILhsfLlcnveTLqNVNkV-ymtSIqNTVINkSiS--gDsdv
SmAChE1   MSygivmNMnLciitsfLLLdpvLssRlNafqNVNNV-lipSIENTIINNSia--aDidL
ShAChE1   MSlgimkNMnyylitsfILLnngLsfKTNTIDNVNNIHlqSSIENTMINNnnSnlvhtdL
SbAChE1   MSlgimkNMnyylitsfLLLnngLsfKTNTIDNVNNIHlqSSIENTMINNnnSnlvhtdL
CsAChE    ---------msgywiayLLLtvtvnf----------------------------------
OvAChE    ---------msgywisyLLLiviasf----------------------------------
SjTAChE   -----------mlftithLfiltLhs---------------------------------I
SmTAChE   --------MlLsfthlsvvslfsIii----------------------------------
ShTAChE   --------MlLsfihlsILslfnMif---------------------------------M
EgAChE    MhkaprpeVrsskmripLssvliLla--------------------------------pt
EmAChE    -------------mripLscvliLlv--------------------------------pt
HsAChE    ---mrppqclLhtpslaspLlllLlw--------------------------------lL
TcAChE    -------mnlLvtsslgvLLhlvvlc----------------------------------
TtAChE    -----------mpyqhpLvLwvpLlc-------------------------------ghL
TsAChE    MTqifkkNkkIttsdklattnrtssv---------NqppfSpldqprqsaSsrieeDehv
SrAChE    ----miirkfycfgnlyLIflviLiw----------------------------------
CbAChE    -------------mrysLLffifLpc----------------------------------
CeAChE    -------------mrnsLLffifLps----------------------------------

SjAChE1   hnektsicssdnPI-tytSvGiycgqrEiVhwpNGpAsiVDvyyGIryAqsPtGSLRFrk
SmAChE1   hndktticssdnPV-VhtSvGiycglrEiVhwpNGpAsmVDvyyGIryAqsPtGSLRFkk
ShAChE1   hndktitclsdnPI-VhtSvGiycglrEiVhwpNGpAsmVDvyyGIryAqsPtGSLRFkk
SbAChE1   hndktitclsdnPI-VhtSvGiycglrEiVhwpNGpAsmVDvyyGIryAqsPtGSLRFkk
CsAChE    -----sdaqdpelI-kqLthGGkvrGqrklvqvdGKqtsinvFhGIPFAqPPVGvrRFAP
OvAChE    -----sdaqdpelI-kqLthGGkvrGqrklvqvdGKqisinvFhGIPFAqPPVGvrRFAP
SjTAChE   ippvkpdiqtyinV--eLShGGtIrGiEetvnidGdefkVnrFLGIPyAkPPIGkLRFAP
SmTAChE   -ipttvksntlkPIEIpLthGGsviGkEeIvnidGQevkVnsFLGIPyAskPIGkLRFAP
ShTAChE   iittnvmlnplkPIKItLnhGGsIiGkEeIvqidGKeiqVnsFLGIPyAskPIGkLRFAP
EgAChE    alasifttqvgnvV-LnLSerssLmGtg--wnisGi--rVDayLGIPFAkPPVGNLRFAP
EmAChE    alasilttqvgnvV-LnLSerssLmGtg--wnisGi--rVDayLGIPFAkPPIGNLRFAP
HsAChE    gggvgaegredael-LvtvrGGrLrGir-lktpgGp---VsaFLGIPFAEPPmGprRFlP
TcAChE    ------qaddhsel-LvntksGkvmGtrvpvlss----hisaFLGIPFAEPPVGNMRFrr
TtAChE    alqkdaptaqltPI-VrLSnGspIrGvE-tvsaNGQ--aVteFLGvPFAEPPIGSfRFrP
TsAChE    gekyngdqqtlsPI-VrLhdGspvrGrl-tysaaGK--mVteFLGIPFAEPPtGrrRFrP
SrAChE    iknskshftstqPl-IyLSdGspIlGqt-llapNGK--iVtqFLGvPFAEPPIGNLRFrk
CbAChE    -------vitavdl-IhLhdGspLfGeE-VlsqtGK--pltrFLGIPFAEPPIGNLRFrk
CeAChE    -------tilavdl-IhLhdGspLfGeE-VlsqtGK--pltrFqGIPFAEPPVGNLRFkk

SjAChE1   PVEpiPepKtIFmAdKLPaSCpQpkDTmFq-NsaAARMWvpNTpMSEDCLfLNIWVPLrE
SmAChE1   PVEpiPepKkIFmAdKLPptCpQpkDTmFq-NsaAARMWvpNTpMSEDCLfLNIWVPiKE
ShAChE1   PVEpiPepKkIFmAdKLPStCpQpkDTmFq-NsaAARMWvpNTpMSEDCLfLNIWVPLKE
SbAChE1   PVEpiPepKkIFmAdKLPStCpQpkDTmFq-NsaAARMWvpNTpMSEDCLfLNIWVPLKE
CsAChE    P-QKhPGWsgIrNATRLPpaCWQYilegFDkNnpAARMWlNNTEMSEDCLYLNIWtP---
OvAChE    P-QKhPGWsgIrNATRLPpaCWQYilegFDkNnpAARMWlNNTEMSEDCLYLNIWtP---
SjTAChE   P-EKhPGWngVrNATnysptCWQYifTgFDVNnpAgKMWiNNTEMSEDCLYLNvWVP---
SmTAChE   P-EKhPGWKgkYNATtLsptCWQYifTgFDavnaAgKMWiNNTEMSEDCLYLNvWtP---
ShTAChE   P-iKhPGWKgLYNATKLsatCWQYifvgFDavnvAgKMWiNNTEMSEDCLYLNvWtP---
EgAChE    PVEadl-WegqldATKLPNSCWQYrpgnFDVtnpAARiWiNNTEMSEDCLYLNvWVP---
EmAChE    PVEadl-WegqldATKLPNSCWQYrpgnFDVtnpAARiWiNNTEMSEDCLYLNvWVP---
HsAChE    PepKqP-WsgVvdATtfqSvCYQYvDTlyp-gFegteMWNpNrElSEDCLYLNvWtP---
TcAChE    PepKkP-WsgVWNAstyPNnCqQYvDeqFp-gFsgseMWNpNrEMSEDCLYLNIWVP---
TtAChE    PkpKaP-WsqVFNATRMPSSCpQsrDvyFE-gFegAeMWNaNTplnEDCLfLNIWVP---
TsAChE    PIsKkP-WteVFeATRqPrSCpQsrDnyFq-NFagAeMWNaNTpldEDCLhLNvWVa---
SrAChE    PLpKkP-WRhVlNATtpPNaCvQslDTyFg-DFfgAdsWNchgplSEDCLYLNIyIP---
CbAChE    PkpKqP-WRipFNATtpPNSCiQseDTyFg-DFygstMWNpNTklSEDCLYLNvyVP---
CeAChE    PkpKqP-WRiplNATtpPNSCiQseDTyFg-DFygstMWNaNTklSEDCLYLNvyVP---
```

```
SjAChE1   SNeshsNSKEkLaVMlWIYGGsFYmGTaTLsVYDaRfLAArqNiIVaSMNYRlGSFGFLY
SmAChE1   SNgshPNSKEkLaVMlWIYGGsFYmGTSTLsVYDaRfLAArqNiIVaSMNYRlGSFGFLY
ShAChE1   SNsshsNSKEkLaVMlWIYGGsFYmGTaTLsVYDaRfLAArqNVIVaSMNYRlGSFGFLY
SbAChE1   SNsshsNSKEkLaVMlWIYGGsFYmGTaTLsVYDaRfLAArqNiIVaSMNYRlGSFGFLY
CsAChE    -syasPt--nlLPVMVWIYGGGytSGTSTLDVYDasVLvAKhkVIVlSMqYRVGAlGFLr
OvAChE    -syasPt--nlLPVMVWIYGGGytSGTSTLDVYDasVLvAKhkVIVlSMqYRVGAlGFLr
SjTAChE   -NntvnD-RrlLPVMVWIYGGGFtSGsanLqVYnGaILsAtqNVIIVSMqYRVGAFGFLr
SmTAChE   ---kssvdsphLPVMVWIYGGGFtSGsanLqVYnGaILsAtqNVIIVSMqYRVGAFGFLr
ShTAChE   ---kpsgknsrfPVMVWIYGGGFtSGsanLqVYnGaILsAtqNVIIVSMqYRVGAFGFLr
EgAChE    --srlqghnasLPVMVWIfGGGFFSGTSTLDVYDGRyLAAmENVIVVSMqYRlGpFGFLF
EmAChE    --srpqghnasLPVMVWIfGGGFFSGTSTLDVYDGRyLAAmENVIVVSMqYRlGpFGFLF
HsAChE    -yprptS---ptPVlVWIYGGGFYSGaSsLDVYDGRfLvqaErtvlVSMNYRVGAFGFLa
TcAChE    -sprpkS----ttVMVWIYGGGFYSGsSTLDVYnGKyLAytEeVvlVSlsYRVGAFGFLa
TtAChE    -grwpPaSRrrLPVMVWvYGGGFWSGTSsLDVYDGKILcsQqdVIIVSMNYRVslFGFLY
TsAChE    -ghqinqrprlLPVMVWvfGGGFWSGTasLDVYDGKILssEEdVvVVSMNYRVslFGFLY
SrAChE    -Neidst--KkLaVlIWIYGGGFWSGcSsLDVYDGKIfAtEENVIIVtlNYRVtvFGFLY
CbAChE    -gkvdPN--KkLaVMIWvYGGGFWSGTSTLDVYDGRILtvEENVIlVaMNYRVsiFGFLY
CeAChE    -gkvdPN--KkLaVMVWvYGGGFWSGTaTLDVYDGRILtvEENVIlVaMNYRVsiFGFLY

SjAChE1   m-----NtEEAP-------------GNMGLwDQrLAmKWikDhIEdFGGDPyriTLFGES
SmAChE1   m-----NtEEAP-------------GNMGLwDQrLAmKWikDhIEhFGGDPyriTLFGES
ShAChE1   m-----NtEEAP-------------GNMGLwDQrLAmKWiknhIEnFGGDPhriTLFGES
SbAChE1   m-----NtEEAP-------------GNMGLwDQrLAmKWikDhIEnFGGDPhriTLFGES
CsAChE    LDPDsGtanplP--NGvPNaNSVArGNqGLLDQqLALeWmHENIaeFGGnPkhVTvFGES
OvAChE    LDPDsGaanplP--NGvPNaNSVArGNqGLLDQqLALeWmHENIaeFGGnPkhVTvFGES
SjTAChE   LDPsiaNnDEmhqnksgsanNSiALGNqGiLDQhMALmWVkENIQrlhGDPnqVTiFGES
SmTAChE   LkPnitdqtqtd-----------ALGNqGLLDQLMALKWVsENIgqFhGDPnqVTiFGES
ShTAChE   LkPnttdqtqts-----------ALGNqGLLDQLMALKWVkENIEqFhGDPdqVTiFGES
EgAChE    v------esqig-------------GNMGLLDQqLALKWVrkhIsaFtGDPglVTLFGES
EmAChE    v------esqig-------------GNMGLLDQqLALKWVrkhIraFtGDPglVTLFGES
HsAChE    Lp----gsrEAP-------------GNvGLLDQrLALqWVqENvaaFGGDPtsVTLFGES
TcAChE    Lh----gsQEAP-------------GNvGLLDQrMALqWVHDNIQfFGGDPktVTiFGES
TtAChE    L-----grpEAP-------------GNvGLLDQLeALKWVHyNIEmFGGDPaeVTLFGES
TsAChE    L-----gladAP-------------GNaGLLDQLeALKWVHrNIaaFGGDPadVTLFGES
SrAChE    m-----grEEAP-------------GNMGLwDQLMAmKWVHkhIEvFGGDPetVTiFGES
CbAChE    m-----NrsEAP-------------GNMGmwDQLLAmKWVHkNIDlFGGDtsriTLFGES
CeAChE    m-----NrpEAP-------------GNMGmwDQLLAmKWVHkNIDlFGGDlsriTLFGES

SjAChE1   AGAVSVStHvvSPwShsYynnAImQSGSiFsnWgLaTsEIsLnqtqrLAKilGC------
SmAChE1   AGAVSVStHvvSPwShsYynnAImQSGSiFsnWgLaTsEVsLnqtqrLAKilGC------
ShAChE1   AGAVSVStHvvSPwShsYynnAImQSGSiFsnWgLaTsEVsLnqtqrLAKilGC------
SbAChE1   AGAVSVStHvvSPwShsYynnAImQSGSiFsnWgLaTsEVsLnqtqrLAKilGC------
CsAChE    sGAVSaSIqWLSPlaQrYFQRvILQSGSaYArWALeSlgeAhiRgqqfAltCGC------
OvAChE    sGAVSaSIqWLSPlaQrYFQRvILQSGSvYArWALdSlgeAhmRgqqfAvaCGC------
SjTAChE   AGAVSiSIlWmSPiaQsYFQRAILQSGSlYArWgLdnaqeAhEKAvefAlaCGC------
SmTAChE   AGAVSVSIlWmSPiaQPYFrRAILQSGSlYArWgLdnaDeAhEKAdvftReCGC------
ShTAChE   AGAVSVSIlWmSPiaQPYFrRAILQSGSlYArWgLdTaDeAhEKAdiftQeCGC------
EgAChE    AGAVSVgLHYLaPsSRslFQRmILQSsSplsrWALwqkpVAhEagisfiKasnC-----a
EmAChE    AGAVSVgLHYLaPsSRslFQRmILQSsSplsrWALwqkpVAhEagisfiKasnC-----a
HsAChE    AGAaSVgmHlLSPpSRglFhRAvLQSGapngPWAtvgmgeArrRAtqLAhlvGC---ppg
TcAChE    AGgaSVgmHiLSPgSRdlFrRAILQSGSpncPWAsvSvaegrrRAveLgRnlnC------
TtAChE    sGAaSVSLHlLSPlSQPYFkRAILQSGSaiAPWAmeSqEtlLERAllLSEaCkC-Nvtir
TsAChE    AGAaSVSLHlLSPlSsPYykRAILQSGaatAPWALenaDVlIERAllLSEaCqC-Nvtir
SrAChE    AGAaSVSmHmLSekStPYFkRAILQSGSatAPWALenrkVALhRvlvvyEhmkCgNiSrn
CbAChE    AGAaSVSIHmLSqkSaPYFhRAIiQSGSatsPWAieprDVALaRAviLynamkC--gNms
CeAChE    AGAaSVSIHmLSPkSaPYFhRAIiQSGSatsPWAieprDVALaRAviLynamkC--gNms
```

```
SjAChE1   -vyPSpsdqIk--CLRSksitEiLDa----------------------------hdtMyd
SmAChE1   -gyrSsndqIk--CLRSksitEiLDa----------------------------hdtMyd
ShAChE1   -gyrSsmdqIk--CLRSksitEiLDa----------------------------hdtMyd
SbAChE1   -gyrSsmdqIk--CLRSksikEiLDa----------------------------hdtMyd
CsAChE    -tsPSvhReaSLkCLqSlhpitLVDnLDSVaefVGqRRrEKLVqlfadsQevlDkskLLn
OvAChE    -tsPSvnReaSLkCLqSlhpitLVDnLDSVaefVGqRRrgKLVqlfedsQkvpDkttLLs
SjTAChE   -ksPSldRkaSLECLqqlDpltLInqLDSINaAIGKRRyNt-VhKclhPttHkNEsfLLg
SmTAChE   -qsPSvdRkaSLECLRklDpltLVnqLDSINvAIGKhRyDa-VrKyllPRyHkqEpfLLs
ShTAChE   -qsPSvdRkaSLECLRklDpltLVnqLDSINvAIGKRRyDt-VkKylrPKnHqrEpfLLs
EgAChE    drlhdlgKeVA--CLRrlpAssvfDtLseltlAstKRRasRLstmssnP-----qWp-VP
EmAChE    drlhdlgKeVA--CLRrlpAssvfDtLseltlAstKRRasRLstmssnP-----qWp-VP
HsAChE    gtggndtelVA--CLRTrpAqvLVnh----------------------------EWhvLP
TcAChE    -nlnSdeelIh--CLRekkpqELIDv----------------------------EWnvLP
TtAChE    drsPnftQlIe--CLlkvpvdELLrn----------------------------EWvtye
TsAChE    drnPdllalVr--CLqqapvsQLLqh----------------------------EWvtye
SrAChE    ldevnmdKvLd--CfmkasAkKiLDs----------------------------EW--sP
CbAChE    lisPdydRiLd--CfqraDAdaLren----------------------------EW--aP
CeAChE    linPdydRiLd--CfqraDAdaLren----------------------------EW--aP

SjAChE1   paS----YFsVPFpPVlDnNFfPyEnSQSF--RQlrhLKpSgaLmfGINKNEGsYFLLYa
SmAChE1   paS----YFsVPFpPVlDnNFfPyEnSQSF--RQlkyLKpSgaLmfGINKNEGsYFLLYa
ShAChE1   paS----YFsVPFpPVlDnNFfPyEnSQSF--RQlkyLKpSgaLmfGINKNEGsYFLLYa
SbAChE1   paS----YFsVPFpPVlDnNFfPyEnSQSF--RQlkyLKpSgaLmfGINKNEGsYFLLYa
CsAChE    waeSsRMYFDVPFkaVVDGyvIPKQPeQmFSPnfhtsLrKSpELLLGVNKNEaMYFLLYG
OvAChE    waeSsRMYFDVPFkaVVDGyvIPKQPeQmFSPnfhtsLrRSpELLLGVNKNEaMYFLLYG
SjTAChE   qstSTRLYFDVPFqPVIDGyvIPKhPdQmFmeyntnasKEkpEiLIGVNeNEaLfFLLpG
SmTAChE   qstSTRLYFDVPlqPVIDGylvPKhPdhiF--nEknkLKQnpELLIGVNtNEaMfFLLpG
ShTAChE   qstSTRLYFDVPlqPVIDGHlMPKhPdQiF--hdknaLKdkpELLIGVNtNEaMfFLLpG
EgAChE    fltdasqYFDVymrPVlDGNFLPDcPgtil--ssissyea-pDvLIGnvaNEGIYwLLYG
EmAChE    fltdasqYFDVymrPVlDGNFLPDcPgtil--ssissyea-pDvLIGnvaNEGIYwLLYG
HsAChE    qeS----vFrfsFvPVVDGDFLsDtPeali---naGdfhg-lQvLVGVvKdEGsYFLvYG
TcAChE    fdS----iFrfsFvPVIDGeFfPtslesml---nsGnfKK-tQiLLGVNKdEGsfFLLYG
TtAChE    -------flDfPwvPVVDhHFvtEEPrall---dsGqfKR-cELLVGnNReEsIYFivYy
TsAChE    -------flDfPwtPVVDhyFLtEQPkall---EsGqfKK-cELLLGsNhdEsIYFivYy
SrAChE    vme----faDfPwvPVIDGDFLvEQaStSl---KeGrfKK-tDLLVGsNqdEaIYFivYq
CbAChE    vre----fgDfPwvPVVDGDFLlEnaqtSl---KqGnfKK-tQLLaGsNRdEsIYFLtYq
CeAChE    vre----fgDfPwvPVVDGDFLlEnaqtSl---KqGnfKK-tQLLaGsNRdEsIYFLtYq

SjAChE1   f--------VtNsKwmKnmTdlpikNrmDyLR--------cLRqVldLDdDD-------r
SmAChE1   f--------VsNsKwmKnlTdlpitNrmDyLR--------cLRqVldLDdDDe------r
ShAChE1   f--------VsNsKwmKnlTdlpitNrmDyLR--------cLRqVldLDdDD-------r
SbAChE1   f--------VsNsKwmKnlTdlpitNrmDyLR--------cLRqVldLDdDD-------r
CsAChE    LAlgNgsFLhEdGtVilPeairlaaarrpqspDgpLADFHrIsSaqfLsEDqLvrGisqL
OvAChE    LAlgNgsFLhEdGsVllPeaiqlaaarrpqspngpLADFHrIsSaqfLsEDqLvrGisqL
SjTAChE   LsIkNtQFLypNGtahmPnsielagNKqsFnKDdELADFfWItttqiLDEshMrlGLAKL
SmTAChE   IAIkdtQFLfsNGsVimPsTmelagkKkpFkEgeEIADFyWItAtqiLDEshMrpGLAKM
ShTAChE   IsIkdtQFLfpNGsVimPdTiglagkKqpFkQgeEIADFyWItAtqiIeEshMrpGLAKL
EgAChE    LgItginFLyENGtVtQPsledlrraKiDyLQ--------lVQtrfms-----------I
EmAChE    LgItginFLyENGtVtQPsledlrraKiDyLQ--------lVQtrfms-----------I
HsAChE    a-----------pgfsKd--nesliSraEFLa-----------gVrvg-----------V
TcAChE    a-----------pgfsKd--seskiSreDFMs-----------gVkls-----------V
TtAChE    v-----------dKIfKr--dhllqNKahFLtDpsL----FeQAVyaL-----------L
TsAChE    v-----------dKIfKr--devf-tKqEFLvDdrL----FeQAVyaL-----------L
SrAChE    L-----------GdIfpP--eeff-vKkEFIKnrEn----WIRSIhnL-----------L
CbAChE    L-----------pdIfpv--adff-SKsEFIKDrQt----WIKgVkdL-----------L
CeAChE    L-----------pdIfpv--adff-tKtDFIKDrQL----WIKgVkdL-----------L
```

```
SjAChE1     PeftePl---iRyTdfEYqnyNqlp--------------TlEsWtERLeeIssDrsFkCP
SmAChE1     PeftePl---iRyTdfEYqtyqqlp--------------TlEsWtERLeeIssDrsFkCP
ShAChE1     PeftePl---iRyTdfEYqtyqqlp--------------TlEsWtERLeeIssDrsFkCP
SbAChE1     PeftePl---iRyTdfEYqtyqqlp--------------TlEsWtERLeeIssDrsFkCP
CsAChE      PSfsyglPvdtiqTtgyaDPsDsrm--------------TaEvlmhRLDtLcGelDFiCP
OvAChE      PSfsyglPvdtsqTngyaDPsDsrm--------------TaEalmhRLDtLcGelDFiCP
SjTAChE     PSyyynlPisssidgyYDPdtvkm--------------sgEdvikRLDefvGDlDFaCP
SmTAChE     PSyyynlPLtsspkrgyYDPdtvyi--------------hdEellrRLDKfaGDlDFaCP
ShTAChE     PSyyynlPLisspkqgyYDPdtihi--------------kdEellrRLDKfaGDlDFaCP
EgAChE      ghllePfP-aiatlqygfNspNipkVTSYNTGLQYNSLTstsaFlDRLDdLsGevDFiCP
EmAChE      ghllePfP-aiatlqygfNspNipeVTSYNTGLQYNSLTstsaFlDRLDdLsGevDFiCP
HsAChE      Pqv---sdLaaeavvlhYtdwlhpe--------------dparlrEaLsdVvGDhnvvCP
TcAChE      Pha---ndLgldavtlQYtdwmddn--------------ngiknrDgLDdIvGDhnviCP
TtAChE      PhkyrknPivyRailfEYmnyElph--------------dplllqDaLDKalGDyhFhCg
TsAChE      PqkyrknPivhRailfEYtdfDrpa--------------TaQrrqqaLDKmfGDyhFtCg
SrAChE      PrqflknsLamsaiihEYEPnslpv--------------kpQsWvDsLDKmlGDfqFtCn
CbAChE      PrqilkcqLtlaavlhEYEPqDlpi--------------saQnWlnamDKmlGDyhFtCs
CeAChE      PrqilkcqLtlaavlhEYEPqDlpv--------------TprdWinamDKmlGDyhFtCs

SjAChE1     TINmAtaVtNDYRIpGrrRAHTLPVYFYEFQHRTlSLPmPKWTGtMHGYEIEYVFGIPFS
SmAChE1     TINmAtaVtNDYRIpGrrRAHTLPVYFYEFQHRTvSLPmPKWTGtMHGYEIEYVFGIPFS
ShAChE1     TINmAtaVtNDYRIpGrrRAHTLPVYFYEFQHRTlSLPmPKWTGtMHGYEIEYVFGIPFS
SbAChE1     TINmAtaVtNDYRIpGrrRAHTLPVYFYEFQHRTlSLPmPKWTGtMHGYEIEYVFGIPFS
CsAChE      TLNFAeqVA---RIansK------VflYEmQrkTlScPfPsWTGVMHGYEIEYVFGMPhS
OvAChE      TLNFAeqVA---RIansK------VflYEmQrkTlScPfPsWTGVMHGYEIEYVFGMPYS
SjTAChE     TLNFAehVA---RLpnaK------VflYhFnkRTnSLPlPKWTGVMHGYEIEYiFGIPYd
SmTAChE     TLNFAeqVA---RLpnaK------VflYhFnkRTeSLPmPKWTGVMHGYEIEYiFGIPYd
ShTAChE     TLNFAeqVA---RLpdaK------VflYhFnkRTeSLPmPtWTGVMHGYEIEYiFGIPhd
EgAChE      TLlFArlLs---KIGGss------VqFYnFiHRTsgctfPEWTGVMHGYEIEYVFGMPFS
EmAChE      TLlFArlLs---KIGGsr------VqFYnFiHRTsgctfPgWTGVMHGYEIEYVFGMPFS
HsAChE      vaqlAgrLA---aqGar-------VYaYvFEHRastLsWPlWmGVpHGYEIEfiFGIPld
TcAChE      lMhFvnkyt---KfGng-------tYlYfFnHRasnLvWPEWmGViHGYEIEfVFGLPlv
TtAChE      vteFArmyq---qyGat-------VYsYyFtHRssqqvWPEWmGavHGYEInfiyGePln
TsAChE      vneFArrfr---dhGsp-------VYsYyFtqRsseqqWPEWmGVlHGYEInYVFGePln
SrAChE      vneFAlahA---ihGGq-------tYYYmFsHRaseqtWPEWmGVlHGYEInfiFGePYn
CbAChE      vnemAlaht---KhGGd-------tfYYyFtHRatqqtWPEWmGVlHGYEInfiFGePFn
CeAChE      vnemAlaht---KhGGd-------tYYYyFtHRasqqtWPEWmGVlHGYEInfiFGePln

SjAChE1     PQFQAsYYrFTDEErQLSdiMMtYWANFArTGDPNilPDGrhvtDneNaEDsDEliEDEf
SmAChE1     PQFQAsFYrFTDEErQLSdiMMtYWANFArTGDPNilPDGrhvtDnlNPdDPDEITEDqL
ShAChE1     PQFQAsFYrFTDEErQLSdiMMtYWANFArTGDPNilPDGrhvtDnvNPEDPDEITggEL
SbAChE1     PQFQAsFYrFTDEErQLSdiMMtYWANFArTGDPNilPDGrhvtDnvNPEDPDEITggEL
CsAChE      ekFQnsFYrFnvEEKKLSdeMMRmWtNFAKrGnPNkndDGtigavv--------------
OvAChE      ekFQnsFYrFnvEEKKLSdeMMRmWtNFAKrGDPNrndDGtigavv--------------
SjTAChE     kEFsrnFYNFTDnEKQLSlRiMKlWsNFAKTGhPsksetGeilqpe--------------
SmTAChE     PEFskqFYNFTDpEKifSsRiMKmWtNFAKTGhPsksnDGkistpe--------------
ShTAChE     PEFskqFYsFTDpEKKfSsRMMemWtNFAKTGhPsksdDGhvsipe--------------
EgAChE      qtFtSKYYNFTNQEaELSlRvMRYWANFAKnGkattdPkGievnEn--------------
EmAChE      qtFtSKYYNFTsQEaELSsRvMRYWANFAKTGkattdPkGievtEn--------------
HsAChE      P-----srNyTaEEKifaqRLMRYWANFArTGDPNeprDpk--apq--------------
TcAChE      -----KelNyTaEEeaLSrRiMhYWAtFAKTGnPNephsqesk-----------------
TtAChE      i----hrYayTEaEKDLSrRfMsYWANFArTGnPNinPDGswtpEl--------------
TsAChE      v----KqfayTEaEKDLarRfMRYWANFArTGnPNvnPDGtwtpEl--------------
SrAChE      t----dkYsyTkEEqELasRfMRYWANFArTGnPNknPDGtftaDn--------------
CbAChE      q----KrfNyTDEErELSnRfMRYWANFAKTGDPNkneDGsftqDi--------------
CeAChE      q----KrfNyTDEErELSnRfMRYWANFAKTGDPNkneDGsftqDv--------------
```

```
SjAChE1    EnSvNlKQrRKNPFIGWPeFRnsTk-----------aYIifrsAP----gnllVGTrPRh
SmAChE1    kDSLsHKQGsKNPFIGWPeFRnsTk-----------aYIvfrsAP----anllVsTrPRh
ShAChE1    EDSLNHKQGRKNPFIGWPeFRnsTk-----------aYIvfrsAP----anllVsTrPRh
SbAChE1    EDSLNHKQGRKNPFIGWPeFRnsTk-----------aYIvfrsAP----anllVsTrPRh
CsAChE     ----------------WPtyKaaTn---rDPALDLDDYIiLd-------tpirrGkdlRQ
OvAChE     ----------------WPayKaakq---rDPALDLDDYIiLd-------tpirrGkdlRQ
SjTAChE    ----------------WPlFRrtdrlVtNDy-----DhfiIe-------dkfkqGSGlRR
SmTAChE    ----------------WPlFhstdgFVSNnP-----DYliLe-------detkLGSGlhR
ShTAChE    ----------------WPlFRrtdgFiSsDP-----DhliLe-------netkLGSGlhR
EgAChE     ----------------WPdFRvtde-----------sYleIgl------ntstVkesPhd
EmAChE     ----------------WPdFRvtde-----------sYleIgl------ntstVkeslhd
HsAChE     ----------------WPpytagaq-----------qYVsLdl------rpleVrrGlRa
TcAChE     ----------------WPlFttkeq-----------kfIdLnt------epmkVhqrlRv
TtAChE     ----------------WPpygkdet------------YliMsa------tqngtGraaRR
TsAChE     ----------------WPkysqdel------------YliLsa------stngtGhGPRR
SrAChE     ----------------WPkytqhTm-----------EYmnLtiesaYangaklIGTGPRR
CbAChE     ----------------WPkynsvSm-----------EYmnMtvesSYP-gqnrIGhGPRR
CeAChE     ----------------WPkynsvSm-----------EYmnMtvesSYP-smkrIGhGPRR

SjAChE1    RQClFWrrwyPaLlQqver----NrQhClgv-----------------------------
SmAChE1    RQClFWrrwyPaLlQqver----NrQhClgv-----------------------------
ShAChE1    RQClFWrrwyPaLlQqver----NrQhClgv-----------------------------
SbAChE1    RQClFWrrwyPaLlQqver----NrQhClgv-----------------------------
CsAChE     vgCrFWlHeIPtLvKerrlldD-kdssCngavtvfssraiivvFiFsvlpavhyivQ---
OvAChE     agCrFWlHeIPtLvKerrsmdD-NgssCndaitifssraiivvifFsvlptihytvQ---
SjTAChE    eRCrFWlHeMsdmtQilrntcq----lsssgiKstgyhhlingFwlflFiqYylye----
SmTAChE    dRCaFWlHeMqdmkdiwfnrcD-----psggiKptgnYililgsglllFigifyg-----
ShTAChE    dRCaFWlHeMkdmtdilndtce-----CssgiKptgsYrfiiglwlllFigYl-------
EgAChE     KgCtFWndifPsLqRiylqrsg-yksYpqsgssavcpYmevdlYpvErlrdFvtgeypDg
EmAChE     KgCtFWngifPtLqRiylqrsg-yksYpqsgssavcpYmevdlYpvErlrdFvtgeypDg
HsAChE     QaCaFWnrfLPkLlsatdtldeaerQWkaefhRw-ssYmvhwknqFDhYskqdrcsdl--
TcAChE     QmCvFWnqfLPkLlnata--cDgelsssgtssskgiifyvlfsilYliF-----------
TtAChE     RQCaFWsNyIPkLhaataslsDmemKWklqmaKwedeYiadwkHhFEmYkrlqqhrylDA
TsAChE     RQCtFWsqyIPkLfaataslsDvemKWkiqmmKweeeYiaewkHhFEmYkkiqqhsymDt
SrAChE     KECsFWkavLPnLitatndvgesvihWrnlmsKweneYivdwqFhFEqYkkYqsyrhsDA
CbAChE     KECaFWkayLPnLmaavadvgDpylvWkqqmdKwqneYitdwqYhFEqYkrYqtyrQsDS
CeAChE     KECaFWkayLPnLmaavadvgDpylvWkqqmdKwqneYitdwqYhFEqYkrYqtyrQsDS

SjAChE1    -------------------
SmAChE1    -------------------
ShAChE1    -------------------
SbAChE1    -------------------
CsAChE     -------------------
OvAChE     -------------------
SjTAChE    -------------------
SmTAChE    -------------------
ShTAChE    -------------------
EgAChE     seRlmlPWVIVfVLLWMLV
EmAChE     seRlmlPWIIVlVLLWMLV
HsAChE     -------------------
TcAChE     -------------------
TtAChE     h--Cggel-----------
TsAChE     y--Crn-------------
SrAChE     ngyCdl-------------
CbAChE     et-Cgg-------------
CeAChE     et-Cgg-------------
```

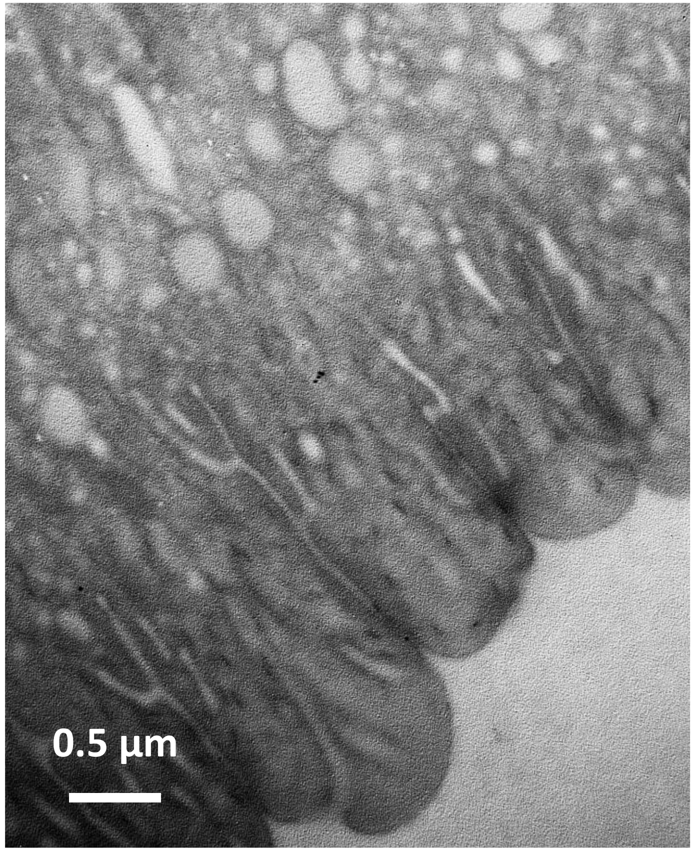

Figure S3

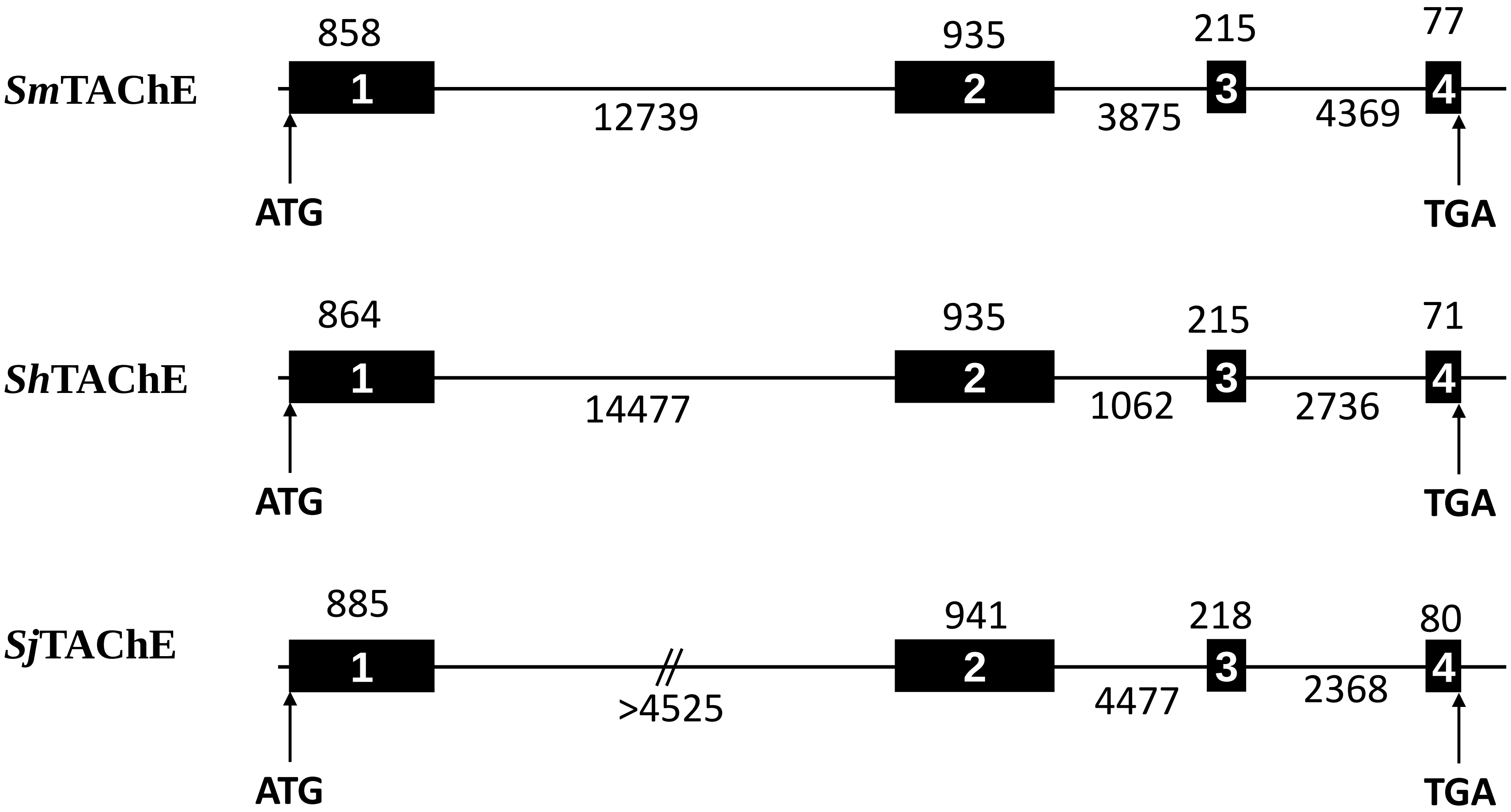

*Sm*TAChE
858
1
12739
935
2
3875
215
3
4369
77
4
ATG
TGA
*Sh*TAChE
864
1
14477
935
2
1062
215
3
2736
71
4
ATG
TGA
*Sj*TAChE
885
1
>4525
941
2
4477
218
3
2368
80
4
ATG
TGA

Figure S4

```
SmTAChE   MLLSFTHLSVVSLFSII--IIPTTVKSNTLKPIEIPLTHGGSVIGKEEIVNIDGQEVKVNSFLGIPYASKPIGKLRFAPPEKHPGWKGKY   88
ShTAChE   MLLSFIHLSILSLFNMIFMIITTNVMLNPLKPIKITLNHGGSIIGKEEIVQIDGKEIQVNSFLGIPYASKPIGKLRFAPPIKHPGWKGLY   90
SjTAChE   MLFTITHLFILTLHSII-----PPVKPDIQTYINVELSHGGTIRGIEETVNIDGDEFKVNRFLGIPYAKPPIGKLRFAPPEKHPGWNGVR   85
          **::: ** :::*..:*        *   :   . *:: *.***:: * ** *:***.*.:** *******. ********** *****:*

SmTAChE   NATTLSPTCWQYIFTGFDAVNAAGKMWINNTEMSEDCLYLNVWTPKSSVDS-PHLPVMVWIYGGGFTSGSANLQVYNGAILSATQNVIIV   177
ShTAChE   NATKLSATCWQYIFVGFDAVNVAGKMWINNTEMSEDCLYLNVWTPKPSGKN-SRFPVMVWIYGGGFTSGSANLQVYNGAILSATQNVIIV   179
SjTAChE   NATNYSPTCWQYIFTGFDVNNPAGKMWINNTEMSEDCLYLNVWVPNNTVNDRRLLPVMVWIYGGGFTSGSANLQVYNGAILSATQNVIIV   175
          ***. * *******.***. * *********************.*: : ..   :***********************************

SmTAChE   SMQYRVGAFGFLRLKPNITDQT-----------QTDALGNQGLLDQLMALKWVSENIGQFHGDPNQVTIFGESAGAVSVSILWMSPIAQP   256
ShTAChE   SMQYRVGAFGFLRLKPNTTDQT-----------QTSALGNQGLLDQLMALKWVKENIEQFHGDPDQVTIFGESAGAVSVSILWMSPIAQP   258
SjTAChE   SMQYRVGAFGFLRLDPSIANNDEMHQNKSGSANNSIALGNQGILDQHMALMWVKENIQRLHGDPNQVTIFGESAGAVSISILWMSPIAQS   265
          *************.*. :::           :: ******:*** *** **.*** ::****:*************:**********

SmTAChE   YFRRAILQSGSLYARWGLDNADEAHEKADVFTRECGCQSPSVDRKASLECLRKLDPLTLVNQLDSINVAIGKHRYDAVRKYLLPRYHKQE   346
ShTAChE   YFRRAILQSGSLYARWGLDTADEAHEKADIFTQECGCQSPSVDRKASLECLRKLDPLTLVNQLDSINVAIGKRRYDTVKKYLRPKNHQRE   348
SjTAChE   YFQRAILQSGSLYARWGLDNAQEAHEKAVEFALACGCKSPSLDRKASLECLQQLDPLTLINQLDSINAAIGKRRYNTVHKCLHPTTHKNE   355
           **:****************.*:****** *:   ***:***:*********::******:*******.****:**::*:* * *  *:.*

SmTAChE   PFLLSQSTSTRLYFDVPLQPVIDGYLVPKHPDHIFNEK--NKLKQNPELLIGVNTNEAMFFLLPGIAIKDTQFLFSNGSVIMPSTMELAG   434
ShTAChE   PFLLSQSTSTRLYFDVPLQPVIDGHLMPKHPDQIFHDK--NALKDKPELLIGVNTNEAMFFLLPGISIKDTQFLFPNGSVIMPDTIGLAG   436
SjTAChE   SFLLGQSTSTRLYFDVPFQPVIDGYVIPKHPDQMFMEYNTNASKEKPEILIGVNENEALFFLLPGLSIKNTQFLYPNGTAHMPNSIELAG   445
           ***.************:******:::*****::* :    *  *::**:***** ***:******::**:****: **:. **.:: ***

SmTAChE   KKKPFKEGEEIADFYWITATQILDESHMRPGLAKMPSYYYNLPLTSSPKRGYYDPDTVYIHDEELLRRLDKFAGDLDFACPTLNFAEQVA   524
ShTAChE   KKQPFKQGEEIADFYWITATQIIEESHMRPGLAKLPSYYYNLPLISSPKQGYYDPDTIHIKDEELLRRLDKFAGDLDFACPTLNFAEQVA   526
SjTAChE   NKQSFNKDDELADFFWITTTQILDESHMRLGLAKLPSYYYNLPISSSSIDGYYDPDTVKMSGEDVIKRLDEFVGDLDFACPTLNFAEHVA   535
          :*: *::.:*:***:***:***::***** ****:********: **   *******: : .*::::***:*.*************:**

SmTAChE   RLPNAKVFLYHFNKRTESLPMPKWTGVMHGYEIEYIFGIPYDPEFSKQFYNFTDPEKIFSSRIMKMWTNFAKTGHPSKSNDGKISTPEWP   614
ShTAChE   RLPDAKVFLYHFNKRTESLPMPTWTGVMHGYEIEYIFGIPHDPEFSKQFYSFTDPEKKFSSRMMEMWTNFAKTGHPSKSDDGHVSIPEWP   616
SjTAChE   RLPNAKVFLYHFNKRTNSLPLPKWTGVMHGYEIEYIFGIPYDKEFSRNFYNFTDNEKQLSLRIMKLWSNFAKTGHPSKSETGEILQPEWP   625
          ***:***********:***:*.****************:* ***::**.*** ** :* *:*::*:***********: *.:  ****

SmTAChE   LFHSTDGFVSNNPDYLILEDETKLGSGLHRDRCAFWLHEMQDMKDIWFNRCDP-SGGIKPTGNYILILGSGLLLFIGIFYG-   694
ShTAChE   LFRRTDGFISSDPDHLILENETKLGSGLHRDRCAFWLHEMKDMTDILNDTCEC-SSGIKPTGSYRFIIGLWLLLFIGYL---   694
SjTAChE   LFRRTDRLVTNDYDHFIIEDKFKQGSGLRRERCRFWLHEMSDMTQILRNTCQLSSSGIKSTGYHHLINGFWLFLFIQYYLYE   707
          **: ** ::::. *::*:*:* * ****:*:** ******.**.:*  : *:  ***** ** : :* *   *:***
```

Figure S5

Table S1. Nucleotide sequences of siRNAs used in RNAi studies.

Target Gene	siRNA Name	siRNA Sequence (5'→3')
**SmAChE1**	SmAC1-siRNA1	ACAATATGTTCGTCTGATAATCCAG
	SmAC1-siRNA2	CTAGAATGTGGGTACCAAATACACC
	SmAC1-siRNA3	CCATCAGGAGCTTTAATGTTTGGCA
**SmTAChE**	SmAC2-siRNA1	CAAACAAGAACCATTCTTATTAAGC
	SmAC2-siRNA2	CTACCACTGTGAAGTCAAATACTCT
**Universal-TAChE***	S-AC2-siRNA	TGAAATGAGTGAAGATTGTTTATAT
**Control**	Cont-siRNA	CTTCCTCTCTTTCTCTCCCTTGTGA

* This siRNA targets the tegumental AChE of the 3 schistosome species examined in this work.

Table S1

Table S2. Nucleotide sequences of oligonucleotides used in RT-qPCR analysis.

Target Gene (Accession No.)	Primer Name	Primer Sequence (5'→3')	Product Size (bp)
**SmAChE1 (AF279461.1)**	SmAC1-F	GATGAAGAACGTCAACTCAGTGATATTAT	96
	SmAC1-R	CACGTGTCGACCATCTGGTAAAATA	
	SmAC1-Probe	FAM-TTTGCACGCACAGGGGAT	
**SmTAChE (OP018961)**	SmAC2-F	CATGAAATGCAAGACATGAAAGATATATGGTT	111
	SmAC2-R	GAAAAGAAGTAACCCAGATCCAAGGAT	
	SmAC2-Probe	FAM-CTGGTGGAATAAAACC	
**Sm-Tubulin-α (M80214.1)**	SmTub-F	TGGTTGACAACGAGGCCATTTAT	68
	SmTub-R	TGTGTAGGTTGGACGCTCTATATCT	
	SmTub-Probe	FAM-TCCGTCGACAAATATC	
**Sm-TPI (M83294.1)**	SmTPI-F	CATACTTGGACATTCTGAGCGTAGA	88
	SmTPI-R	ACCTTCAGCAAGTGCATGTTGA	
	SmTPI-Probe	FAM-CAATAAGTTCATCAGATTCAC	

Table S2

Table S3. Quantitative summary of relative AChE activity (fold difference) displayed by live *S. mansoni* (***Sm***), *S. haematobium* (***Sh***) and *S. japonicum* (***Sj***) adults. *S. mansoni* AChE activity value is set at 1. For total lysate assays, 30 µg of total protein were used per assay.

**Live Parasites**		**Total Lysate**	
**Live Male Parasites**		**Male Lysate**	
Sm	1	***Sm***	1
Sh	2.30 ± 0.54	***Sh***	2.05 ± 0.01
Sj	0.78 ± 0.25	***Sj***	0.52 ± 0.12
**Live Female Parasites**		**Female Lysate**	
Sm	1	***Sm***	1
Sh	6.53 ± 0.81	***Sh***	8.40 ± 0.95
Sj	1.05 ± 0.07	***Sj***	0.49 ± 0.07
**Male/Female**		**Male/Female**	
Sm	2.38 ± 0.32	***Sm***	1.79 ± 0.14
Sh	0.85 ± 0.21	***Sh***	0.47 ± 0.01
Sj	1.72 ± 0.45	***Sj***	2.17 ± 0.34
